# A pragmatic parallel arm randomized-controlled trial of a multi-pronged electronic health record-based clinical decision support tool protocol to reduce low-value antipsychotic prescriptions among older adults with Alzheimer’s and related dementias

**DOI:** 10.1371/journal.pone.0277409

**Published:** 2022-12-20

**Authors:** John N. Mafi, Anne M. Walling, Chad Villaflores, Sitaram Vangala, Andrea Sorensen, Eric Cheng, Ashley Turner, Zoe Trutner, Grace Cheng, Julia Cave Arbanas, Benjamin Waterman, Suzanne Shu, Noah Goldstein, Catherine Sarkisian

**Affiliations:** 1 Division of General Internal Medicine and Health Services Research, David Geffen School of Medicine at UCLA, Los Angeles, California, United States of America; 2 RAND Corporation, Santa Monica, California, United States of America; 3 VA Greater Los Angeles Healthcare System, Los Angeles, California, United States of America; 4 Division of Geriatrics, David Geffen School of Medicine at UCLA, Los Angeles, California, United States of America; 5 Division of Medical Informatics, David Geffen School of Medicine at UCLA, Los Angeles, California, United States of America; 6 David Geffen School of Medicine at UCLA, Los Angeles, California, United States of America; 7 Division of Marketing, Charles H. Dyson School of Applied Economics and Management at Cornell University, Ithaca, New York, United States of America; 8 Division of Management and Organizations, Anderson School of Management at UCLA, Los Angeles, California, United States of America; Shahid Beheshti University of Medical Sciences, ISLAMIC REPUBLIC OF IRAN

## Abstract

Among patients with Alzheimer’s disease and its related dementias (ADRD) with behavioral disturbances, antipsychotic prescriptions have limited efficacy and increase the risk of death. Yet, physicians continue to routinely prescribe low-value antipsychotic medications for behavioral disturbances among patients with ADRD. We designed a pragmatic randomized-controlled trial to measure the impact of a behavioral economic electronic health record (EHR) clinical decision support (CDS) intervention to reduce physician prescriptions of new antipsychotic medications among patients with ADRD. Utilizing a pragmatic parallel arm randomized-controlled trial design, the study will randomize eligible physicians from a large academic health system to either receive a EHR CDS intervention or not (control) when they prescribe a new antipsychotic medication during visits with patients with ADRD. The intervention will include three components: 1) alerts prescribers that antipsychotic prescriptions increase mortality risk (motivating physicians’ intrinsic desire for non-malfeasance); 2) offers non-pharmacological behavioral resources for caregivers; 3) auto-defaults the prescription to contain the lowest dose and number of pill-days (n = 30) without refills if the prescriber does not cancel the order (appealing to default bias). Over 1 year, we will compare the cumulative total of new antipsychotic pill-days prescribed (primary outcome) by physicians in the intervention group versus in the control group. The study protocol meets international SPIRIT guidelines. Behavioral economics, or the study of human behavior as a function of more than rational incentives, considering a whole host of cognitive and social psychological preferences, tendencies, and biases, is increasingly recognized as an important conceptual framework to improve physician behavior. This pragmatic trial is among the first to combine two distinct behavioral economic principles, a desire for non-malfeasance and default bias, to improve physician prescribing patterns for patients with ADRD. We anticipate this trial will substantially advance understanding of how behavioral-economic informed EHR CDS tools can potentially reduce harmful, low-value care among patients with ADRD.

## Introduction

Among persons with Alzheimer’s disease and its related dementias (ADRD), disruptive behaviors such as agitation are frequent. In these challenging clinical situations, physicians commonly prescribe antipsychotic medications as a first-line treatment, despite limited evidence for their efficacy in ADRD, and extensive empiric data showing their association with over-sedation, cognitive worsening, falls, strokes, and even death [1–3]. Such a treatment epitomizes low-value care, defined as patient care that offers no net benefit in specific clinical scenarios. While the benefits of antipsychotics as a first-line therapy for patients with ADRD may justify the risks in a small fraction of clinical scenarios, there is a strong consensus that overall prescribing rates are too high, while effective multidisciplinary and nonpharmacological approaches are underutilized [4, 5]. There is also broad consensus among physician and patient stakeholders both within and beyond the medical community that reducing low-value prescribing of antipsychotic medications for adults with ADRD is an important priority for quality of life improvement and mortality reduction [4, 5]. Despite this broad consensus as well as an FDA 2005 “black box” warning discouraging the use of antipsychotics in this population, physicians continue to frequently prescribe these medications as a first-line therapy for behavioral disturbances among patients with ADRD [6, 7].

Physician biases and a “more is better” cultural mindset play important roles in medical decision-making; whether and to what extent cognitive biases should be leveraged to reduce low-value prescribing is an area of active research [8–11]. Previous studies have evaluated behavioral economic approaches to reducing low-value antipsychotic prescribing, such as peer comparison letters to high prescribers [12]. More broadly, behavioral economists have described “default bias” as a preference for the current state of affairs, and value-based care researchers have successfully leveraged this bias by implementing EHR clinical decision support (CDS) tools that default to generic prescriptions over brand-name equivalents [13, 14]. Perhaps the most powerful cognitive motivator of physician behavior is the notion of physician non-malfeasance (do no harm), which has been championed by physicians since at least the time of Hippocrates in 421 B.C.E. [15]. The power of non-malfeasance as a motivator of physician behavior was re-demonstrated in a recent randomized trial of clinical vignettes, which illustrated that physicians are more motivated to reduce low-value prescribing when financial penalties to hospitals are framed in terms of patient harm rather than societal harms or increased health care costs [16].

In this context, the study team proposes a parallel arm randomized controlled trial (RCT) to understand how multidisciplinary teams can combine behavioral insights including principles of non-malfeasance and preference for the current state of affairs (default bias) through an EHR CDS tool to reduce harmful antipsychotic prescriptions in adults with ADRD. Furthermore, with most health systems nationwide using the Epic EHR and the Epic “App Orchard” that facilitates shared innovation, the intervention–if successful–could be easily and widely disseminated. Broad implementation of this intervention has the potential to meaningfully decrease falls, strokes, and mortality for patients with ADRD in the United States.

## Methods

### Study aims

The goal of this study is to design, implement, and test the impact of a quality improvement (QI) intervention that uses an EHR CDS tool among physicians newly ordering an antipsychotic medication for adults with ADRD to increase guideline-concordant prescribing. The study team hypothesizes that the intervention will reduce participating clinicians’ pill days per patient prescribed.

### Study design and setting

**Fig 1:**
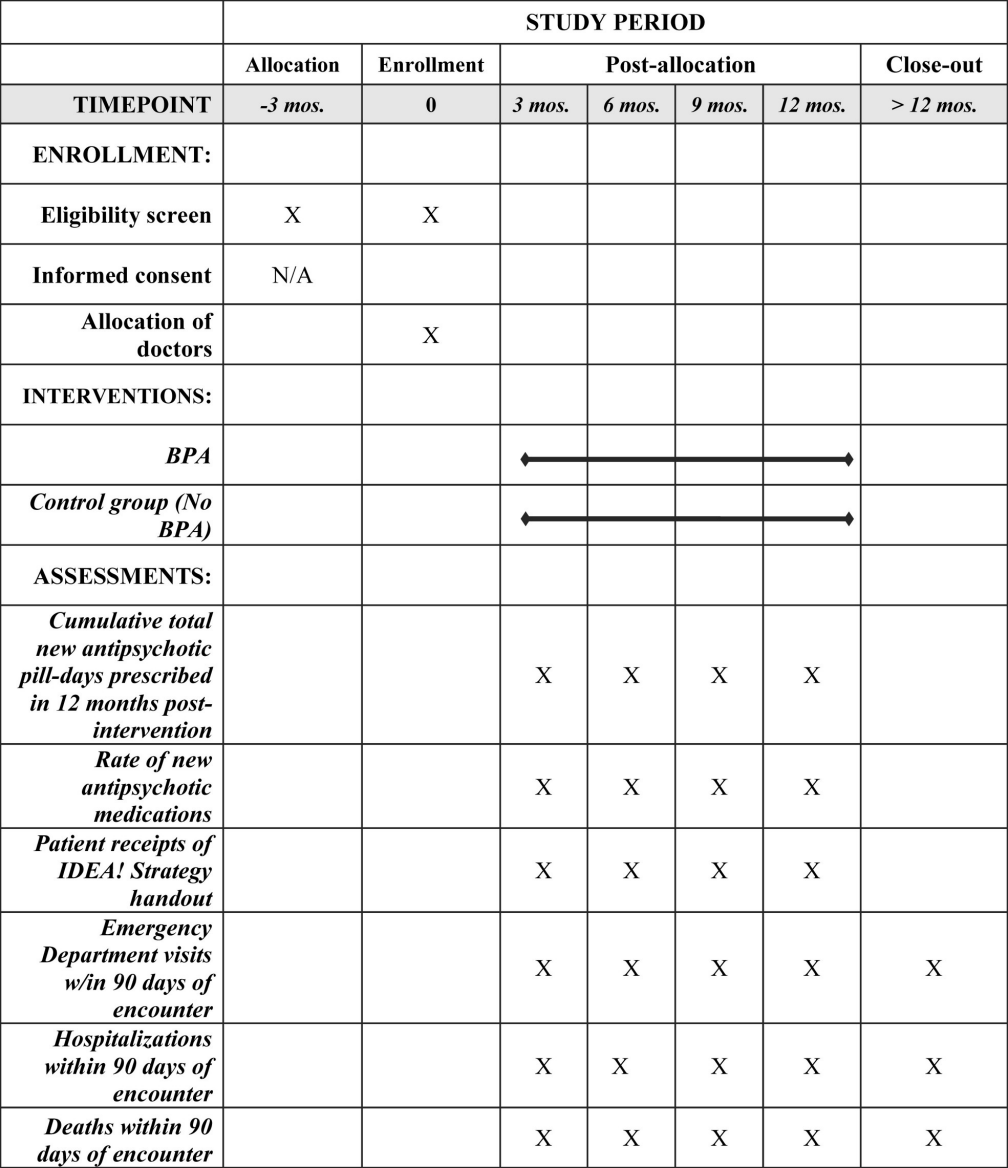
SPIRIT Schedule of enrollment, interventions, and assessments. Although all eligible physicians are allocated into either the intervention or control groups, physicians are only enrolled in the trial once they prescribe a new antipsychotic medication to an eligible patient, which could occur at any point within the 12-month study period. Hospitalizations include psychiatric hospitalizations.

This study design is a pragmatic parallel arm randomized-controlled trial. The study team will randomize eligible physicians (see below for eligibility criteria) at UCLA Health, a large academic health system in Los Angeles, California, to be either exposed to the EHR CDS tool (intervention) or not (control) in a 1:1 allocation ratio over a 12-month period when they initiate a prescription for a new antipsychotic medication during a visit with a patient with ADRD (Fig 1).

### Ethical considerations

The Institutional Review Board (IRB) of the University of California, Los Angeles (UCLA) approved this protocol on 01/27/2020 (IRB#19–002122). The IRB and National Institute on Aging (NIA) Data Safety Monitoring Board (DSMB) approved a waiver of informed consent because this trial is a part of ongoing quality and patient safety improvement efforts. In addition, the Principal Investigator will notify all of the study participants that this project is an NIH-funded clinical trial and provide them with the trial registration link after the study has been completed. This study has been registered in ClinicalTrials.gov, with trial registration identifier: NCT04851691.

The study data containing patient health information will be safeguarded with firewalls and encryption following UCLA IRB guidelines, as well as a unique study ID created to protect the identities of patients. If successful, UCLA plans to adopt full integration of the EHR CDS tool for all eligible clinicians.

### Inclusion criteria

Eligible physicians for the EHR CDS tool intervention include physicians who provide ambulatory care in the UCLA health system and have generated a new antipsychotic prescription (e.g., Quetiapine, Olanzapine, Risperidone, Aripiprazole, Haloperidol, Clozapine) for eligible patients (described below) in the UCLA health system at least once between 1/1/2019-4/30/2021 (n = 149). Based on current prescribing patterns at UCLA, we estimate that the vast majority of physicians in the study (>75%) will only see this EHR CDS tool 1–2 times over a year, suggesting little impact on clinical workflow and lessening opportunities for contamination. Eligible physicians will be enrolled in the study during their first encounter with one of the patients (see above) during which a new antipsychotic medication order is initiated.

Inclusion criteria for patients will include: 1) having an assigned primary care physician (PCP) and/or assignment to an Accountable Care Organization (ACO) at UCLA Health, and 2) being part of the health system’s EHR-based dementia registry. The health system’s dementia registry uses a select group of ICD-10 codes included in the patient’s problem list to identify patients with ADRD, without age restrictions (Table 1). This ICD-based approach was implemented by the health system as an update to a previously published approach [17]. To measure the specificity of this method for correctly classifying patients as having ADRD, physicians on the study team (board-certified geriatrician, palliative care specialist, and primary care general internist) conducted an implicit review (e.g., expert clinician diagnostic judgment) [18] on a random sample of 30 registry patients; 29 out of 30 (97%) had ADRD.

**Table 1:** Dementia international classification of diseases 9^th^ and 10^th^ revision (ICD-9 and ICD-10) codes.

	ICD-9 codes	ICD-10 codes
At least one code from a problem list	42, 46.11, 46.19, 46.79, 49.9, 94.9, 199.1, 277.39, 290, 290.1, 290.11, 290.12, 290.13, 290.2, 290.21, 290.3, 290.4, 290.41, 290.42, 290.43, 290.8, 290.9, 291.2, 292.82, 293, 294.1, 294.11, 294.2, 294.21, 294.8, 294.9, 296.9, 297.9, 298.9, 305.9, 310.2, 311, 319, 323.9, 330.1, 330.8, 331, 331.1, 331.11, 331.19, 331.4, 331.5, 331.6, 331.82, 331.83, 331.9, 332, 332.1, 333, 333.4, 340, 345.9, 348.1, 349.9, 369.9, 437, 440.9, 459.9, 781, 781.3, 784.3, 797, 907, 999.9, E980.5, V17.2, V40.31	A52.17, A81.00, A81.01, A81.9, A86, B20, C80.1, E75.6, E85.4, F01, F01.5, F01.50, F01.51, F02.80, F02.81, F03, F03.9, F03.90, F03.91, F05, F06.8, F07.81, F10.27, F10.97, F13.27, F13.97, F18.17, F18.97, F19.17, F19.97, F22, F32.9, F39, F84.2, G10, G20, G21.8, G23.1, G30, G30.0, G30.1, G30.8, G30.9, G31.01, G31.09, G31.83, G31.84, G31.85, G31.9, G35, G40.909, G91.2, G91.9, G93.1, G98.8, H54.7, I67.2, I67.3, I67.850, I68.0, I70.90, IMO0002, R25.2, R27.0, R41.0, R41.81, R47.01, S06.9X9S, T80.89XA, Z91.83

Consistent with prior research [19, 20], patients will be excluded from eligibility if they have diagnosis codes for schizophrenic disorders, delusion disorders, bipolar disorders, or other non-organic psychoses on their problem list (Table 2). Patients with Parkinson’s disease on their problem list will also be excluded because quetiapine is clinically indicated to decrease hallucinations induced by dopaminergic medications in patients with Parkinson’s disease [21]. Additionally, because the target for this intervention will be new prescriptions, if patients have been prescribed antipsychotics in the prior 12 months, they will not be eligible. Eligible encounters will include ambulatory office visits and scheduled telephone and video telehealth visits. Emergency department visits, observational stays, and inpatient hospitalizations will not be eligible for the CDS to fire.

**Table 2:** ICD-9 and ICD-10 codes for exclusion criteria reflecting severe mental illness.

Diagnosis Code Type	Diagnosis Code
ICD-9-CM	295
ICD-9-CM	295.1
ICD-9-CM	295.2
ICD-9-CM	295.3
ICD-9-CM	295.4
ICD-9-CM	295.5
ICD-9-CM	295.6
ICD-9-CM	295.7
ICD-9-CM	295.8
ICD-9-CM	295.9
ICD-9-CM	296
ICD-9-CM	296.1
ICD-9-CM	296.4
ICD-9-CM	296.5
ICD-9-CM	296.6
ICD-9-CM	296.7
ICD-9-CM	296.8
ICD-9-CM	297.1
ICD-9-CM	297.2
ICD-9-CM	297.3
ICD-9-CM	297.8
ICD-9-CM	298.3
ICD-9-CM	298.4
ICD-10-CM	F31.9
ICD-10-CM	F22
ICD-10-CM	F24
ICD-10-CM	F23
ICD-10-CM	F52.8
ICD-10-CM	F31.89
ICD-10-CM	F34.0
ICD-10-CM	F09
ICD-10-CM	F06.1
ICD-10-CM	F31.70
ICD-10-CM	F43.0
ICD-10-CM	G30.9
ICD-10-CM	F02.80

### Intervention

**Fig 2:**
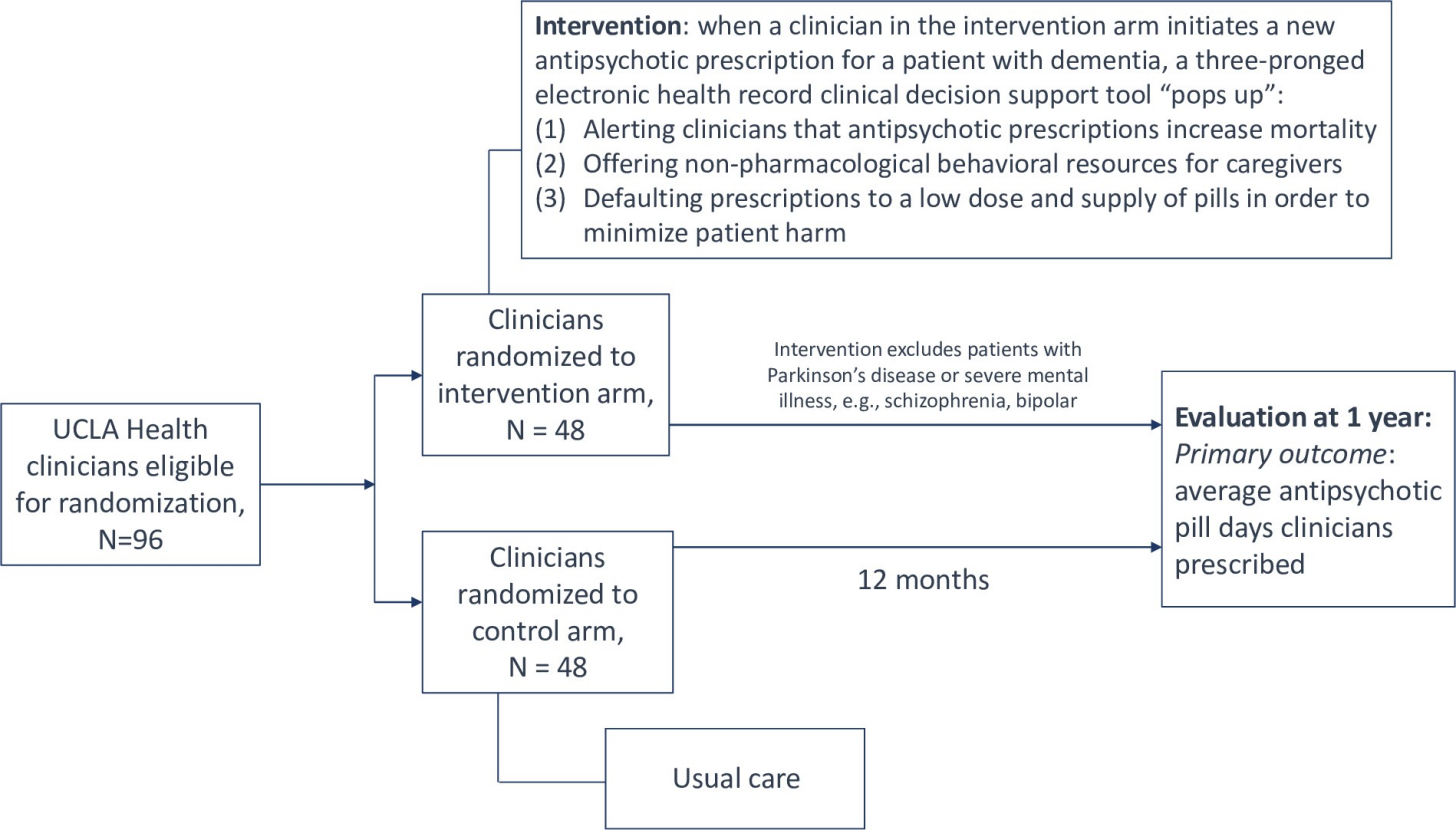
EHR CDS tool design. EHR = electronic health records; CDS = clinical decision support.

We applied theoretically grounded behavioral economic methods to design an EHR CDS to reduce low-value antipsychotic prescriptions in adults with ADRD at UCLA Health. The EHR CDS tool (Fig 2) was designed with input from a multidisciplinary team including a geriatrician, a palliative care specialist, a primary care general internist, a quality officer, informatics specialists, and two behavioral economists. The study team previously presented the proposed intervention in an iterative fashion to several stakeholder teams including physicians, informaticists, and health system leaders. The final intervention was approved by the UCLA Health System Primary Care Council (which includes patient representation), Alert Committee, and the Ambulatory Operations Advisory Group.

The intervention was designed based on a growing body of scientific evidence that multicomponent interventions are more likely to succeed than single-component interventions to reduce low-value care [22]. The intervention (Fig 2) is an EHR CDS that will include three components: 1) evidence from Choosing Wisely^TM^ guidelines that encourage the clinician to avoid prescribing antipsychotics by highlighting increasing patient mortality risk and thereby appealing to the physician’s desire for non-malfeasance; 2) prompts to incorporate IDEA! strategy resources on how caregivers can best manage a patient’s behavioral disturbance non-pharmacologically [23], which will be available in the EHR to include in the patient’s after visit summary; and 3) for physicians who do not cancel their prescription order, an automatic default to a low dosing and low number of pill days prescribed in the order set (30 pill-days). The study team consulted a pharmacist expert (GC) on appropriate defaults for antipsychotics in terms of dosing, frequency, and number of pills supplied. For example, currently in the UCLA EHR quetiapine defaults to 25 mg by mouth once daily 90 tabs x 3 refills, totaling 360 pill-days. The study team will change the default to 25 mg by mouth once daily 30 tabs with no refills, totaling 30 pill-days. Physicians will be free to increase amounts as they desire and thus this EHR CDS will not include a “hard stop.” The study team opted against the hard stop in acknowledgement of the clinical complexity of this vulnerable patient population and in recognition of the fact that an outright ban on such prescriptions may have unintended consequences including but not limited to endangerment of caretakers or cohabitants. The EHR CDS tool will fire when the eligible physician places a new antipsychotic order for an eligible patient. Once the CDS tool fires and the physician places the order for either the original dose or a lower dose, the patient becomes no longer eligible due to having an active antipsychotic order. Therefore, it will not fire for prescription renewals.

### Intervention implementation

We designed and pilot tested the EHR CDS in the informatics laboratory, which is led by the Chief Medical Informatics Officer. The CDS tool was also pilot tested among the research team, to assess correct activation and smooth functionality. The study team specifically designed the intervention to seamlessly fit within the EHR to minimize workflow disruption by only adding one additional click if accepting the intervention to avoid prescribing, and two clicks if rejecting the intervention in order to place the order. All UCLA Health clinicians will be alerted via email (through the usual mechanism to highlight new EHR tools at UCLA Health System) to the availability of the IDEA! handout that can be used for post-visit instructions to support non-pharmaceutical approaches to ADRD behavioral symptoms. The study team will pilot test the intervention to run silently in the EHR background just prior to activation in order to ensure proper activation of the EHR CDS tool. Just before the onset of the EHR intervention, the Principal Investigator (CS, geriatrician) will send a notification email to all physicians in the intervention arm to “prime” the physicians by notifying them they prescribed antipsychotics for patients with dementia in the past year, informing them of the upcoming EHR CDS rollout, and highlighting non-pharmacologic alternatives including the IDEA! handout [23].

### Randomization & blinding

A statistician not involved in data collection will randomize each eligible clinician to the intervention or control group; physicians will remain in the same arm of the study throughout the entire study period. The randomization of clinicians will be stratified by the number of new antipsychotic prescriptions during the baseline year: 1 vs. 2–3 vs. 4 or more new prescriptions. The randomization of the clinicians was incorporated into the EHR, tagging the providers as either EHR Intervention or EHR Control in a list created for this intervention. The study team will randomize physicians rather than patients to minimize contamination. Contamination occurs when a physician sees a patient in the intervention arm at one time point, learns from the intervention that ordering antipsychotic medications is wrong and/or difficult, and then avoids prescribing these medications in the future, even when seeing a patient in the control arm. Because physicians will on average receive the intervention 1–2 times during the study period, inter-physician contamination will be minimal. Moreover, the CDS tool will only trigger for a new antipsychotic order, defined as an antipsychotic prescription for a patient who has not had an active antipsychotic prescription within the previous 12 months. This intervention will be implemented as part of a quality improvement effort; clinicians will not be blinded to receiving the intervention.

## Study outcomes

### Primary outcome

The primary outcome will be the cumulative total of new antipsychotic prescription days supplied by clinicians per eligible patient in the 12 months after the intervention rollout date compared to the prior 12-months, in the intervention versus control groups regardless of the number of visits the provider receives.

### Secondary outcomes

Secondary outcome measures will be compared between the intervention and control groups and will include the rate of new initiation of antipsychotic medications, patient receipt of the non-pharmacologic IDEA! Strategy handout in post-visit patient instruction, emergency department visits, hospitalizations (including psychiatric hospitalizations), and death within 90 days after the encounter. If patients are seen in the ED or hospitalized for falls within 90 days of being exposed to the intervention, the study team will conduct an implicit medical record review to determine whether these outcomes were unintended consequences of the intervention, considering that patients may have seen other physicians either outside the study or in a different study arm after the initial encounter. The study team will examine whether the intervention led to any unintended consequences such as substitution of other psychotropic medications or reduced time living at home (due to residence in a nursing home or other non-home institution). The study team will also survey clinicians’ perceptions of changes in workflow, autonomy, satisfaction, and quality of care.

### Data collection methods

Information sources will include: 1) administrative data found in the UCLA EHR; 2) survey data collected from clinicians in the intervention group; and 3) manual medical record review for patients with ED, falls, or death within 90 days of intervention exposure to assess for potential unintended consequences (Table 3).

**Table 3:** Study outcome measures.

Domains	Measures	Data Sources	Unit of Analysis
Primary Clinical	Mean antipsychotic pill days (across all patients)	EHR	Clinician
Secondary Clinical	Number of new antipsychotic prescriptions	EHR	Patient
	Receipt of IDEA strategy handout	EHR	Patient
	ED visits	EHR	Patient
	Hospitalizations	EHR	Patient
	All-cause mortality	EHR	Patient
	Fall-related ED visit or hospitalization	EHR, medical record review	Patient
	High value new antipsychotic presciptions for those with severe mental illness	EHR	Patient
	Patient-level average antipsychotic pill days	EHR	Patient
	Patient level new antipsychotic prescriptions	EHR	Patient

### Statistical analyses

**Fig 3:**
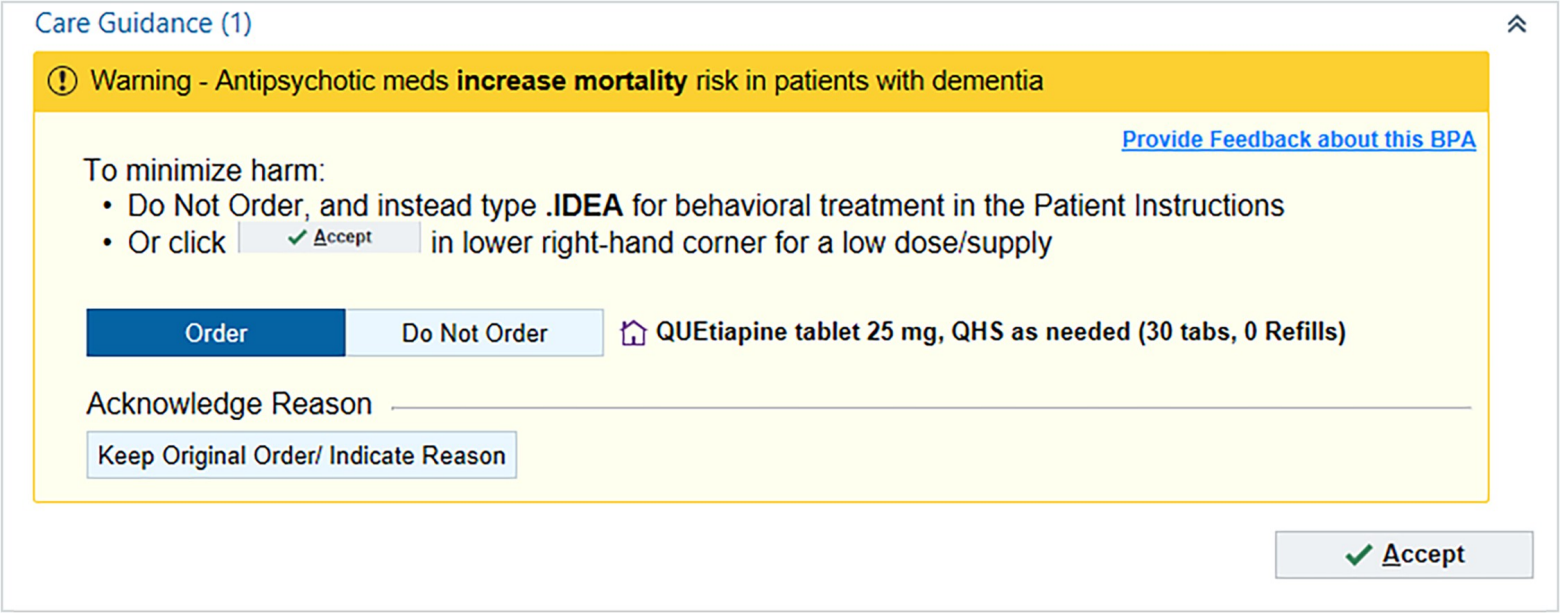
CONSORT flow diagram.

The study protocol follows SPIRIT international guidelines and will follow CONSORT guidelines when reporting the trial results (Fig 3). The complete trial protocol can be accessed in the supporting information files. Specifically, the study team will report descriptive statistics to characterize the sample of patients and clinicians included in the study. The study team will review patient charts to display the time series of new antipsychotic prescriptions during the 12 months before intervention rollout, and the 12 months after rollout. Each patient will be monitored for 90 days after their encounter with a physician in either the intervention or control arm, so the follow up period could last up to 90 days after the completion of the trial. The analysis of the primary outcome–total prescription pill-days–will utilize a physician-level linear regression model, with heteroscedasticity-robust standard errors, following prior published approaches [12]. The model will include a fixed effects study arm (intervention vs. control) and baseline number of new prescriptions, adjusting for physician-level characteristics including physician gender and specialty. Analysis of secondary outcomes will proceed similarly, with the sensitivity analyses using different distributions and link functions as appropriate (e.g., a Bernoulli distribution with a logit link for the death outcome). To ensure equal consideration of outcomes among both groups, the study team will include 90-day follow-up for each outcome measure. A p-value of less than 0.05 will be considered statistically significant. All analyses will follow the intention-to-treat principle and will be performed using SAS v. 9.4 (SAS Institute Inc., Cary, NC).

### Sample size

Power was evaluated using a simulation study. In each simulation, a data set was constructed, combining actual prescribing data from mid-2019 through mid-2020 with simulated data from the subsequent year. The number of total pill days per clinician in the subsequent year was simulated to preserve the mean and standard deviation across clinicians from the prior year, with an assumed year-to-year correlation within clinicians of 0.90. We have previously observed that a small number of providers do a large amount of prescribing, and a large number of prescribers do very little prescribing. The small number of physicians who are likely to continue their high levels of prescribing, leading to high correlation. Clinicians were then randomized into intervention and control arms, with the intervention arm’s number of pill days reduced by a constant amount. A linear regression of year 2’s number of pill days was fitted with study arm and year 1’s number of pill days as predictors. Heteroskedasticity-robust standard errors were used to compute p-values. Significance was defined as a p-value less than 0.05 evaluating the study arm effect in the model, and power was estimated as the percentage of simulations in which a significant difference was obtained. We performed 10,000 simulations to estimate power. Based on historical data, we estimate that about 65% of physicians included in our randomization list will see an eligible patient during the study period. We thus expect our analytic sample to include data from 96 physicians. With 48 physicians randomized to each arm, we will have 80% power to detect a mean reduction of 108 pill days, which is feasible. This simulation power analysis was performed using R v. 3.6.2 (http://www.r-project.org/).

## Discussion

Our study will assess whether a multi-pronged EHR CDS intervention alerting physicians to patient harm, encouraging non-pharmacologic approaches, and defaulting to a lower dose and supply of pills can reduce low-value antipsychotic prescriptions among adults with ADRD. This study is the first to the study team’s knowledge to combine two behavioral economic approaches into a single intervention to improve quality of care among patients with ADRD: appealing to physicians’ desire to avoid malfeasance and auto-defaulting to the lowest supply possible. The study team anticipates that the intervention will reduce low-value antipsychotic prescriptions.

Using the physician surveys, the study team also anticipate that the intervention will provide important preliminary insights into how physician beliefs, preferences, and psychological tendencies can influence the probability of delivering low-value care and the probability of engaging with the EHR CDS intervention.

Limitations to the study include that this will be a single-center setting and may not be generalizable to other settings. Additionally, the three-component intervention design will make it difficult to isolate the most important component of the intervention. Specifically, we will not be able to delineate the effects of the CDS tool from the effects of behavioral economic principles. Albeit, due to the challenges of changing physician behavior, there is growing interest in enhancing CDS’ effectiveness by applying behavioral economic principles. There will also be a possibility of intervention contamination among control physicians, though the study team will attempt to mitigate this by only notifying physicians randomized to the intervention arm about the intervention. Finally, an important caveat is that informed and engaged caregivers and a strong social/family network of support are a mainstay of high-quality ADRD care [5]. While our intervention will provide important caregiver resources and education materials, it cannot of course replace this mainstay of support, which may not always be readily available.

Ultimately, this pragmatic trial will advance understanding of how EHR CDS defaults can reduce harmful, low-value care among adults with ADRD and could inform future interventions across a broader array of conditions and patient populations.

## Supporting information

S1 File SPIRIT checklist. (PDF)

S2 File CONSORT checklist. (PDF)

S3 File Study protocol documentation. (PDF)

S1 Data (XLSX)
