# Correction: A pragmatic parallel arm randomized-controlled trial of a multi-pronged electronic health record-based clinical decision support tool protocol to reduce low-value antipsychotic prescriptions among older adults with Alzheimer’s and related dementias

**DOI:** 10.1371/journal.pone.0315516

**Published:** 2024-12-05

**Authors:** John N. Mafi, Anne M. Walling, Chad Villaflores, Sitaram Vangala, Andrea Sorensen, Eric Cheng, Ashley Turner, Zoe Trutner, Grace Cheng, Julia Cave Arbanas, Benjamin Waterman, Suzanne Shu, Noah Goldstein, Catherine Sarkisian

There is an error in Tables 1 and 2. The ICD-9 codes should have been excluded from Tables 1 and 2. Please see the correct Tables 1 and 2 here.

**Table 1:** Dementia international classification of diseases 10th revision (ICD-10) codes

	ICD-10 Codes
At least one code from a problem list	G30.1, G30.0, G30.8, G30.9, F03.90, F05, F01.50, F01.51, F10.97, F10.27, F19.97, F02.80, F02.81, F03 and all related subcodes, F03.90, F03.91

**Table 2:** ICD-10 codes for exclusion criteria reflecting severe mental illness

Diagnosis Code Type	Diagnosis Code
ICD-10-CM	F31.9
ICD-10-CM	F22
ICD-10-CM	F24
ICD-10-CM	F23
ICD-10-CM	F52.8
ICD-10-CM	F31.89
ICD-10-CM	F34.0
ICD-10-CM	F09
ICD-10-CM	F06.1
ICD-10-CM	F31.70
ICD-10-CM	F43.0
ICD-10-CM	G30.9
ICD-10-CM	F02.80
