# Supplementary material for: A pragmatic parallel arm randomized-controlled trial of a multi-pronged electronic health record-based clinical decision support tool protocol to reduce low-value antipsychotic prescriptions among older adults with Alzheimer’s and related dementias
Source: PLoS One. 2022 Dec 20;17(12):e0277409. doi: 10.1371/journal.pone.0277409 (PMC9767350; doi:10.1371/journal.pone.0277409)
Supplement: S3 File — (PDF) [file pone.0277409.s003.pdf]

An Electronic Clinical Decision Support Tool to Reduce Low-value Antipsychotic Prescriptions

The safety and scientific validity of this study is the responsibility of the study sponsor and investigators. Listing a study does not mean it has been evaluated by the U.S. Federal Government. Read our disclaimer for details.

ClinicalTrials.gov Identifier: NCT04851691

Recruitment Status : Enrolling by invitation
First Posted : April 20, 2021
Last Update Posted : June 24, 2022

Sponsor:
University of California, Los Angeles

Collaborator:
National Institute on Aging (NIA)

Information provided by (Responsible Party):
Catherine A. Sarkisian, University of California, Los Angeles

Study Details | Tabular View | No Results Posted

Study Description

Go to

Brief Summary:

The goal of this study will be to design, implement and test the impact of a quality improvement (QI) intervention that uses an EHR CDS tool among physicians newly ordering an antipsychotic medication for older adults with ADRD. The study team hypothesizes that the intervention will reduce each participating clinician's pill days per patient prescribed.

Condition or disease	Intervention/treatment	Phase
Alzheimer Disease<br>Dementia Alzheimers<br>Dementia of Alzheimer Type	Behavioral: EHR CDS<br>Other: Usual Care	Not Applicable

Detailed Description:

Importance: Among patients with Alzheimer's disease and its related dementias (ADRD) with behavioral disturbances, antipsychotic prescriptions have limited efficacy and substantially increase risk of death. Despite an FDA 2005 "black box" warning and multiple professional physician society guidelines discouraging their use, physicians continue to frequently prescribe antipsychotic medications as first-line therapy for behavioral disturbances among patients with ADRD.

Objective: This study will measure the impact of a multi-pronged electronic health record (EHR) clinical decision support (CDS) tool intervention to reduce physician prescriptions of new antipsychotic medications among older adults with ADRD.

Design, Setting, and Participants: Utilizing a pragmatic parallel cluster-randomized trial design, the study will randomize eligible physicians from a large urban academic medical center to either receive an EHR CDS tool (intervention) or not (control) when they prescribe a new antipsychotic medication during a visit with a patient with ADRD. The intervention will include three components: (1) alerts the prescriber that antipsychotic prescriptions increase mortality; (2) offers non-pharmacological behavioral resources for caregivers; and if the prescriber does not cancel the order (3) auto-defaults the prescription to contain the lowest dose and number of pills (n=30) without refills. In addition, the PI will email all providers randomized to the intervention arm in order to make them aware of the components of this intervention and its motivation. Acknowledging the clinical complexity of this vulnerable patient population, the multidisciplinary study team attempted to design the intervention to maximize impact while minimizing clinician burden. Over a one-year timeframe, the study team will compare the cumulative total of new antipsychotic pill-days prescribed (primary outcome) by physicians in the intervention group versus in the control group.

Hypothesis: This pragmatic trial will advance understanding of how a multi-pronged EHR CDS tool can potentially reduce harmful, low-value care among older adults with ADRD.

Study Design

Go to

Study Type :

Interventional (Clinical Trial)

Estimated Enrollment ℹ :
117 participants

Allocation:
Randomized

Intervention Model:
Parallel Assignment

Masking:
Single (Participant)

Primary Purpose:
Health Services Research

Official Title:
A Pragmatic Randomized-controlled Trial of a Multi-pronged Electronic Health Record-based Clinical Decision Support Tool to Reduce Low-value Antipsychotic Prescriptions Among Older Adults With Alzheimer's and Related Dementias

Actual Study Start Date ℹ :
August 3, 2021

Estimated Primary Completion Date ℹ :
December 23, 2022

Estimated Study Completion Date ℹ :
December 30, 2023

Resource links provided by the National Library of Medicine

MedlinePlus Genetics related topics: Alzheimer disease

MedlinePlus related topics: Dementia

Genetic and Rare Diseases Information Center resources: Familial Alzheimer Disease

U.S. FDA Resources

Arms and Interventions

Go to

Arm ℹ	Intervention/treatment ℹ

Arm	Intervention/treatment
Experimental: EHR CDS tool<br>Multi-pronged electronic health record (EHR) clinical decision support (CDS) tool intervention to reduce physician prescriptions of new antipsychotic medications among older adults with ADRD	Behavioral: EHR CDS<br>When a clinician initiates a new antipsychotic prescription for a patient with dementia, a three-pronged electronic health record clinical decision support tool "pops up": (1) Alerting clinicians that antipsychotic prescriptions increase patient mortality; (2) Offering non-pharmacological behavioral resources for caregivers via a link to the IDEA! strategy resources on how caregivers can best manage a patient's behavioral disturbance non-pharmacologically, which will be available in the EHR to include in the patient's after visit summary; and (3) Defaulting prescriptions to a low supply of pills in to order to minimize harm. In addition, the PI will email all providers randomized to the intervention arm in order to make them aware of the components of this intervention and its motivation.
Experimental: Control<br>Physicians will not receive intervention and perform duties as usual.	Other: Usual Care<br>Patients will receive usual care from their physicians.

Outcome Measures

Go to

Primary Outcome Measures :

1. Cumulative total of new antipsychotic pill-days prescribed [Time Frame: 12 month time point]

   Cumulative total of new antipsychotic prescription days supplied by clinicians per eligible patient in the 12 months after the intervention rollout date compared to the prior 12-months

Secondary Outcome Measures :

1. Number of patients who receive handout [Time Frame: 12 month time point]

Number of patients who receive the non-Pharmacologic IDEA Strategy handout at 12 month time point comparing the intervention vs. control

2. Number of patients with ER visit [Time Frame: 90 days]

   Number of patients with at least one emergency department visit within 90 days of being exposed to the intervention

3. Number of patients with hospitalization [Time Frame: 90 days]

   Number of patients with at least one hospitalization within 90 days of being exposed to the intervention

4. Hospitalizations (including psychiatric hospitalizations) [Time Frame: 90 days]

   Hospitalizations (including psychiatric hospitalizations) within 90 days of being exposed to the intervention

5. Death within 90 days after enrollment [Time Frame: 90 days]

   Death within 90 days after enrollment

Eligibility Criteria

Go to

Information from the National Library of Medicine

Choosing to participate in a study is an important personal decision. Talk with your doctor and family members or friends about deciding to join a study. To learn more about this study, you or your doctor may contact the study research staff using the contacts provided below. For general information, Learn About Clinical Studies.

Ages Eligible for Study:
18 Years and older (Adult, Older Adult)

Sexes Eligible for Study:
All

Accepts Healthy Volunteers:
No

Criteria

Inclusion Criteria:

- Physicians who will be eligible to receive the EHR CDS tool intervention include those who provide ambulatory care in the UCLA health system and have newly prescribed antipsychotics (e.g., Quetiepine, Olanzapine, Risperidone, Aripiprazole, Haloperidol, Clozapine) for eligible patients (described below) at the medical center in the last 24 months.
- Eligible patients will be enrolled in the study during their first encounter with one of the randomized physicians (see above) during which a new antipsychotic medication order will be initiated. Inclusion criteria for patients will include: 1) having an assigned primary care physician (PCP) and/or assignment to an Accountable Care Organization (ACO) at UCLA Health, and: 2) being part of the health system's EHR-based dementia registry.

Exclusion Criteria:

- Patients who have diagnosis codes for schizophrenic disorders, delusion disorders, bipolar disorders, or other non-organic psychoses on their problem list.
- Patients with Parkinson's disease on their problem list
- Patients who have been prescribed antipsychotics in the prior 12 months

Contacts and Locations

Go to

Information from the National Library of Medicine

To learn more about this study, you or your doctor may contact the study research staff using the contact information provided by the sponsor.

Please refer to this study by its ClinicalTrials.gov identifier (NCT number): ***NCT04851691***

Locations

United States, California

UCLA Health
Los Angeles, California, United States, 90095

Sponsors and Collaborators

University of California, Los Angeles

National Institute on Aging (NIA)

Investigators

Principal Investigator: Catherine A Sarkisian, MD, MSPH University of California, Los Angeles

More Information

Go to

Publications:

Schneider LS, Tariot PN, Dagerman KS, Davis SM, Hsiao JK, Ismail MS, Lebowitz BD, Lyketsos CG, Ryan JM, Stroup TS, Sultzer DL, Weintraub D, Lieberman JA; CATIE-AD Study Group. Effectiveness of atypical antipsychotic drugs in patients with Alzheimer's disease. N Engl J Med. 2006 Oct 12;355(15):1525-38.

Hwang YJ, Dixon SN, Reiss JP, Wald R, Parikh CR, Gandhi S, Shariff SZ, Pannu N, Nash DM, Rehman F, Garg AX. Atypical antipsychotic drugs and the risk for acute kidney injury and other adverse outcomes in older adults: a population-based cohort study. Ann Intern Med. 2014 Aug 19;161(4):242-8. doi: 10.7326/M13-2796.

Maher AR, Maglione M, Bagley S, Suttorp M, Hu JH, Ewing B, Wang Z, Timmer M, Sultzer D, Shekelle PG. Efficacy and comparative effectiveness of atypical antipsychotic medications for off-label uses in adults: a systematic review and meta-analysis. JAMA. 2011 Sep 28;306(12):1359-69. doi: 10.1001/jama.2011.1360. Review. Erratum in: JAMA. 2012 Jan 11;307(2):147.

American Geriatrics Society. Ten things clinicians and patients should question. Choosing wisely: an initiative of the ABIM foundation. http://www.choosingwisely.org/societies/american-geriatrics-society/. Accessed Revised April 23, 2015.

Reus VI, Fochtmann LJ, Eyler AE, Hilty DM, Horvitz-Lennon M, Jibson MD, Lopez OL, Mahoney J, Pasic J, Tan ZS, Wills CD, Rhoads R, Yager J. The American Psychiatric Association Practice Guideline on the Use of Antipsychotics to Treat Agitation or Psychosis in Patients With Dementia. Am J Psychiatry. 2016 May 1;173(5):543-6. doi: 10.1176/appi.ajp.2015.173501.

Kuehn BM. FDA warns antipsychotic drugs may be risky for elderly. JAMA. 2005 May 25;293(20):2462.

Maust DT, Strominger J, Bynum JPW, Langa KM, Gerlach LB, Zivin K, Marcus SC. Prevalence of Psychotropic and Opioid Prescription Fills Among Community-Dwelling Older Adults With Dementia in the US. JAMA. 2020 Aug 18;324(7):706-708. doi: 10.1001/jama.2020.8519.

Morgan DJ, Leppin AL, Smith CD, Korenstein D. A Practical Framework for Understanding and Reducing Medical Overuse: Conceptualizing Overuse Through the Patient-Clinician Interaction. J Hosp Med. 2017 May;12(5):346-351. doi: 10.12788/jhm.2738. Review.

Colla CH. Swimming against the current--what might work to reduce low-value care? N Engl J Med. 2014 Oct 2;371(14):1280-3. doi: 10.1056/NEJMp1404503.

Mafi JN, Parchman M. Low-value care: an intractable global problem with no quick fix. BMJ Qual Saf. 2018 May;27(5):333-336. doi: 10.1136/bmjqs-2017-007477. Epub 2018 Jan 13.

Bourdeaux CP, Davies KJ, Thomas MJ, Bewley JS, Gould TH. Using 'nudge' principles for order set design: a before and after evaluation of an electronic prescribing template in critical care. BMJ Qual Saf. 2014 May;23(5):382-8. doi: 10.1136/bmjqs-2013-002395. Epub 2013 Nov 26.

Davidai S, Gilovich T, Ross LD. The meaning of default options for potential organ donors. Proc Natl Acad Sci U S A. 2012 Sep 18;109(38):15201-5. Epub 2012 Sep 4.

Patel MS, Day SC, Halpern SD, Hanson CW, Martinez JR, Honeywell S Jr, Volpp KG. Generic Medication Prescription Rates After Health System-Wide Redesign of Default Options Within the Electronic Health Record. JAMA Intern Med. 2016 Jun 1;176(6):847-8. doi: 10.1001/jamainternmed.2016.1691.

Sacarny A, Barnett ML, Le J, Tetkoski F, Yokum D, Agrawal S. Effect of Peer Comparison Letters for High-Volume Primary Care Prescribers of Quetiapine in Older and Disabled Adults: A Randomized Clinical Trial. JAMA Psychiatry. 2018 Oct 1;75(10):1003-1011. doi: 10.1001/jamapsychiatry.2018.1867.

Roddy E, Jones E. On Hippocrates. Hippocratic ideals are alive and well in 21st century. BMJ. 2002 Aug 31;325(7362):496.

Liao JM, Schapira MS, Navathe AS, Mitra N, Weissman A, Asch DA. The Effect of Emphasizing Patient, Societal, and Institutional Harms of Inappropriate Antibiotic Prescribing on Physician Support of Financial Penalties: A Randomized Trial. Ann Intern Med. 2017 Aug 1;167(3):215-216. doi: 10.7326/L17-0102. Epub 2017 Jun 20.

Schpero WL, Morden NE, Sequist TD, Rosenthal MB, Gottlieb DJ, Colla CH. For Selected Services, Blacks And Hispanics More Likely To Receive Low-Value Care Than Whites. Health Aff (Millwood). 2017 Jun 1;36(6):1065-1069. doi: 10.1377/hlthaff.2016.1416.

Seppi K, Ray Chaudhuri K, Coelho M, Fox SH, Katzenschlager R, Perez Lloret S, Weintraub D, Sampaio C; the collaborators of the Parkinson's Disease Update on Non-Motor Symptoms Study Group on behalf of the Movement Disorders Society Evidence-Based Medicine Committee. Update on treatments for nonmotor symptoms of Parkinson's disease-an evidence-based medicine review. Mov Disord. 2019 Feb;34(2):180-198. doi: 10.1002/mds.27602. Epub 2019 Jan 17. Review. Erratum in: Mov Disord. 2019 May;34(5):765.

Colla CH, Morden NE, Sequist TD, Schpero WL, Rosenthal MB. Choosing wisely: prevalence and correlates of low-value health care services in the United States. J Gen Intern Med. 2015 Feb;30(2):221-8. doi: 10.1007/s11606-014-3070-z. Epub 2014 Nov 6. Erratum in: J Gen Intern Med. 2016 Apr;31(4):450.

Responsible Party:

Catherine A. Sarkisian, Principal Investigator, University of California, Los Angeles

ClinicalTrials.gov Identifier:

NCT04851691 History of Changes

Other Study ID Numbers:

R01AG059815-01S1 (U.S. NIH Grant/Contract)

1R01AG059815-01 (U.S. NIH Grant/Contract)

R01AG059815-01S1 (U.S. NIH Grant/Contract)

First Posted:

April 20, 2021 Key Record Dates

Last Update Posted:

June 24, 2022

Last Verified:

June 2022

Studies a U.S. FDA-regulated Drug Product:

No

Studies a U.S. FDA-regulated Device Product:

No

Keywords provided by Catherine A. Sarkisian, University of California, Los Angeles:

Low Value Care

Antipsychotic medication

Additional relevant MeSH terms:

Alzheimer Disease

Dementia

Brain Diseases

Central Nervous System Diseases

Nervous System Diseases

Tauopathies

Neurodegenerative Diseases

Neurocognitive Disorders

Mental Disorders

Study Title and Key Personnel

All items marked with a red asterisk (*) are required. Items without an asterisk may or may not be required depending on whether the items are applicable to this study.

1.0 ***Full Title of the Submission:**
Pragmatic Trial of an Electronic Health Record Intervention to Reduce Anti-psychotic Medications Among Older Adults with ADRD

1.1 **Protocol Version Date and/or Number:**

2.0 ***Working or Lay Title:**
Reducing Prescribing of Antipsychotics among ADRD Patients

3.0 **Principal Investigator:**

3.1 ***Name:** CATHERINE SARKISIAN
Degree(s): If degrees are not shown here, please add them to the next section, Section 1.1a/Item 1.0, which will then update the Principal Investigator's webIRB account information.
MD, MSHS

3.2 **UCLA Title:**

3.3 ***Will the Principal Investigator conduct the informed consent process with potential study participants?**

- ○ Yes
- ○ No
- ◉ **Not Applicable**

3.4 ***Is the Principal Investigator an undergraduate student, graduate student, post-doctoral fellow, or resident physician?**
○ Yes ◉ **No**

3.4.1 **If you answered "yes" to the above question, indicate the Faculty Sponsor for this study.**

3.5 **UCLA Policy 900 defines types of UCLA employees who may be eligible to serve as a Principal Investigator. Check the policy to see if the Principal Investigator for this study needs an exception to the eligibility requirements.**

If an exception is needed, either attach the letter of exception here, or indicate a Faculty Sponsor in the above item.

Document Name	Document Version #
There are no items to display	

4.0 **Study Contact Person: Indicate the person, in addition to the Principal Investigator, who should receive**

all of the study correspondence.
NADIA AKRAM

5.0 **List the key personnel and study staff below.**

Note: All personnel listed below are required to complete CITI training courses (except for Fund Managers and Regulatory Coordinators). Please verify CITI training completion for all personnel prior to submitting a New Study application or Amendment application to add personnel. Verify using the Training Log tab in the application workspace (accessible by clicking the Exit button at the bottom of this page). HIPAA training is also required if personnel will be accessing protected health information.
Please make sure to have all personnel update their webIRB profile and contact information. Instructions on how to update the webIRB profile are available here.

	Name	Department	Role	Other Role (if applicable)	Will Obtain Consent?	Manage device accountability?	Access to personally identifiable info?	Access to code key?
View	NADIA AKRAM	MEDICINE-GERIATRICS	Study Coordinator		no	Not Applicable	No	No
View	MIGUEL CUEVAS	MEDICINE-GENERAL MEDICINE & HLTH SRVCS.	Research Assistant		no	Not Applicable	No	No
View	Katherine Dominguez	MEDICINE-GENERAL MEDICINE & HLTH SRVCS.	Statistician or Data Analyst<br>Data Manager		no	Not Applicable	Yes	Yes
View	JOHN MAFI	MEDICINE-GENERAL MEDICINE & HLTH SRVCS.	Co-Investigator		no	Not Applicable	Yes	Yes
View	ANDREA SORENSEN	MEDICINE-GERIATRICS	Study Coordinator		no	Not Applicable	No	No
View	Chad Wes Villaflores	MEDICINE-HEMATOLOGY-ONCOLOGY	Data Manager		no	Not Applicable	Yes	Yes
View	ANNE WALLING	MEDICINE-GENERAL MEDICINE & HLTH SRVCS.	Co-Investigator		no	Not Applicable	Yes	Yes

ID: IRB#19-002122 View: NEW 1.1a - Other Personnel

This view has been locked by amendment(s)

Other Personnel

All items marked with a red asterisk (*) are required. Items without an asterisk may or may not be required depending on whether the items are applicable to this study.

1.0 **Principal Investigator**

1.1 **Name:** CATHERINE SARKISIAN
*__Please type the Degree(s):__ MD, MSHS

1.2 **Principal Investigator's UCLA Department:** MEDICINE-VA WADSWORTH MED CTR

1.3 ***Protocol's UCLA Home Department:** MEDICINE-VA WADSWORTH MED CTR

This response defaults to the PI's payroll department. If you wish to affiliate this protocol with another department, please select the department from the list above.

For tips on effective search, please see guidance to the right.

2.0 **If there will be other types of personnel working directly under the PI's supervision on aspects of the study, provide their name, title and institution, indicate their responsibilities, training and qualifications and complete Item 2.1.**

Please also indicate, if applicable, whether that person will obtain consent, manage device accountability, have access to personally identifiable information and/or have access to the code key.

Please use a new entry to add each individual unless describing a class of individuals who rotate through the study team (see guidance area to the right).

Note: If there will not be other types of personnel go to Item 3.0.

Name, title, institution	Study role(s): e.g., conduct interviews/surveys, recruit participants, obtain consent, review records, etc.
There are no items to display	

For existing protocols: Item 2.0 has been modified and this item cannot be edited. When submitting an amendment please use the information found in the text box below to complete Item 2.0 above.
Briefly describe the other study personnel.

2.1 **Indicate the human subjects research training these personnel have or will receive. If training is required in a language other than English or if research is occurring in a location where research personnel do not have access to the internet (e.g., rural community without internet capability), please describe how human subjects training requirements will be fulfilled.**

Check all that apply:

- ☐ CITI Training
- ☐ UC HIPAA Training
- ☐ Other

2.2 **If you indicated "Other" to item 2.1, describe:**

2.3 ***Will this study use the UCLA Health Sciences Volunteer Program to assist with the conduct of the research study?**

○ Yes ● **No**

3.0 ***Will any of the study procedures or analyses be contracted to a consultant or an organization?**

○ Yes ● **No**

3.1 **If yes, specify the consultant(s) and/or organization(s) and the work that they will do for the study.**

ID: IRB#19-002122 View: NEW 1.1b - Type of Study Review

This view has been locked by amendment(s)

Type of Study Review

1.0 ***Indicate the level of risk involved with this study.**

(if there are multiple groups or phases associated with this study, select the highest level of risk.)

- (•) **Minimal risk or no known risks - Click here for the OHRPP tip sheet on minimal risk.**
- ( ) Greater than minimal risk

2.0 ***Indicate the type of review that you are requesting for this study.**

- (•) **IRB Review: Expedited or Full Board**
- ( ) Certification of Exemption from IRB Review

2.1 **If you indicated "IRB Review: Expedited or Full Board" as the type of review in item 2.0, select the IRB that you think best matches your research.**

	Name	Description
( )	Medical Institutional Review Board 1	MIRB1 reviews general and internal medicine, infectious diseases and ophthalmologic research.
( )	Medical Institutional Review Board 2	MIRB2 reviews oncology and hematology research.
( )	Medical Institutional Review Board 3	MIRB3 reviews neuroscience, neurology, psychiatric, drug abuse and dental research.
( )	North General Institutional Review Board	NGIRB reviews research from the College of Letters & Science and the Professional Schools.
(•)	**South General Institutional Review Board**	SGIRB reviews social-behavioral research from the Schools of Public Health, Nursing, and Medicine.

Please note*: *The above requests are for initial routing purposes only. The final decision as to committee assignment and type of review, rests with OHRPP and/or the IRBs.

3.0 ***Is this a COVID-19 research proposal that falls under the following scope:**

a. **Access to the suspected and confirmed UCLA Health COVID-19 patients.**
b. **Access to the electronic medical record chart or data of those patients.**
c. **Access to the remnant or research biospecimen collection of those patients.**
d. **Planning any clinical research interventional trial (drug/device) for those patients.**
e. **COVID Population-based studies that overlap the UCLA Health population or UCLA healthcare workers.**

- ( ) Yes
- (•) **No**

ID: IRB#19-002122 View: NEW 1.2 - Conflict of Interest Information

Conflict of Interest Information

1.0 * **Does the Principal Investigator, any of the key personnel, or their spouses, registered domestic partners, or dependent children, have a financial interest in the sponsor (profit, non-for-profit) of the research?**

○ Yes ◉ **No**

1.1 **If yes, attach a completed copy of the Financial Interests Form for each person who indicates a financial or related interest:**

Document Name	Document Version #
There are no items to display	

2.0 * **Does the Principal Investigator, any of the key personnel, or their spouses, registered domestic partners, or dependent children, have any financial interests related to the research sponsored by a government agency?**

○ Yes ◉ **No**

2.1 **If yes, attach a completed copy of the Financial Interests Form:**

Document Name	Document Version #
There are no items to display	

3.0 * **Indicate whether any of these financial interests have been submitted to or reviewed by the UCLA campus Conflict of Interest Review Committee (CIRC):**

○ Yes ◉ **No**

3.1 **If you have received a response from CIRC, attach it here:**

Document Name	Document Version #
There are no items to display	

ID: IRB#19-002122 View: NEW 1.3 - Study Locations

Study Locations

1.0 ***Indicate the locations where any research activities will be performed by the UCLA research team with participants and/or private information obtained.**

Check all that apply:

- [x] **a. UCLA Sites or UCLA Health System Sites (Does not include Harbor-UCLA Medical Center, Olive View-UCLA Medical Center, or Orthopaedic Institute for Children)**
- [ ] b. Off Campus (in California)
- [ ] c. Outside California (in the U.S.)
- [ ] d. Outside the United States ***See note at right**
- [ ] e. Internet

1.1 **If you selected b, c or d above, please provide your assurance that documentation of each site's permission to conduct the research at the site(s) will be obtained and maintained by the UCLA PI as applicable:**

Agree ☐

2.0 *****Is this a multi-institutional study (i.e., a collaborative project with other sites that have their own IRBs or principal investigators)?**
(Includes but not limited to UC MOU and CTSI MOU collaborations where UCLA IRB review is requested.)

○ Yes ◉ **No**

If no, please skip directly to the next page, do not complete the questions below.
If yes, please answer items 2.1-2.3:

2.1 **Will UCLA be responsible for the overall direction of the study at the other institutions?**

○ Yes ○ No

2.1.1 **Indicate the measures that will be taken to assure regulatory compliance at each site and that the following types of information will be communicated to the other sites: study procedures; modifications to the protocol and related documents; and safety updates, interim results and other information that may impact risks to study participants.**

Check all that apply:

☐ Conference calls or meetings with minutes distributed to each site
☐ Timely e-mail communications
☐ Postings on the study website
☐ Other

2.1.1.1 **If you chose "other", describe.**

2.1.2 **If you answered "yes" to item 2.1 above, please provide your assurance that the current IRB approval for each site(s) will be obtained and maintained by the UCLA PI as applicable:**

Agree ☐

2.2 **Will the UCLA principal investigator specified on this application be responsible for the data coordinating center?**

2.3 **Indicate the anticipated total number of study participants that will be enrolled across all of the institutions.**

ID: IRB#19-002122 View: NEW 2.1 - Project Identification Information

Project Identification Information

1.0 ***Type of Submission (Select one)**

- (●) **Research Study**
- ( ) Application for Approval of "Research Participant Pool" or recruitment database only

2.0 ***Type of Submission (Select one)**
For Amendments, do not undo the response below. Undoing the response may remove sections of the original application.

- (●) **New Submission**
- ( ) Transfer of Ongoing Research from Another Site from Investigator moving to UCLA. Please complete Item 2.1.

2.1 **If you selected "Transfer of Ongoing Research" in Item 2.0 indicate the current status of the study and a brief summary of the work to date.**

3.0 ***Who developed this study?**

Check all that apply:

- [x] **UCLA investigator**
- [ ] Investigator from another institution
- [ ] Industry/Pharmaceutical Company
- [ ] Cooperative Group (e.g., Children's Oncology Group, AIDS Clinical Trial Group)
- [ ] Other

3.1 **If other, specify.**

4.0 **Review For and Reliance Upon External IRBs.**

***Indicate if one of the following applies to this study. (Select one)**

- (●) **None of the options apply.**
- ( ) UCLA IRB to serve as IRB of record for another institution.
- ( ) UCLA to RELY on another IRB.
  This includes reliance using UC MOU, CTSI, NCI, RAND, and Western IRBs.

5.0 ***Is this study cancer related,** including the recruitment of individuals with cancer, collection of cancer human biological samples, specimens or data, or the recruitment of individuals because they are cancer survivors or at risk of developing cancer?

( ) Yes (●) **No**

Note: If you answered "Yes", you must submit an application to the Jonsson Comprehensive Cancer Center (JCCC) Internal Scientific Peer Review Committee (ISPRC). Click here for instructions for submitting to the ISPRC. The ISPRC approval notice or letter of exemption should be attached in Section 2.1/Item 7.2 of the webIRB application.

6.0 ***Nurse Involvement:** Does this study involve any nursing time, effort, and/or resources at UCLA Health System sites, including as subjects, investigators, clinical care providers or data or specimen collectors?

( ) Yes (●) **No**

Note: If you answer "Yes", please submit an application to the Research and Innovation Council (RIC) (formerly

Nursing Practice Research Council (NPRC)). For contact information or for more information about RIC and how to apply, click here. ***IRB approval is not contingent on RIC approval and you do not need to upload documentation of approval from the RIC into webIRB.***

7.0 ***Federal regulations (45 CFR 46.111) require scientific review before an IRB approves a study. For the majority of studies being reviewed and approved by the UCLA IRB, the IRB performs this review.**
See http://ora.research.ucla.edu/OHRPP/Documents/Policy/4/Scientific_Review.pdf for additional details.

Do you want the IRB to consider external scientific or scholarly review?

(●) **Yes** ( ) No

7.1 **If yes, indicate the source of scientific or scholarly review for the study.**

Check all that apply.

- [x] **National Institutes of Health (NIH)**
- [ ] The funding agency (other than NIH)
- [ ] Faculty Sponsor
- [ ] JCCC – Internal Scientific Peer Review Committee (ISPRC)
- [ ] Clinical Translational Research Center (CTRC)
- [ ] UCLA Department
- [x] **Other**

7.1.1 **If you checked "other", describe.**
This IRB is a supplement to an existing study for which we received IRB approval. As such, we have attached the NIH Summary Statement for the parent grant.

Full Title of Study: Pragmatic Trial of an Electronic Health Record/Behavioral Economics Intervention to Reduce Pre-Operative Testing for Cataract Surgery
Protocol ID: IRB#18-001240

7.2 **Attach a copy of the scientific or scholarly review, if applicable.**

Document Name	Document Version #
NIH_Summary Statement	0.01

ID: IRB#19-002122 View: NEW 2.2 - Lay Summary and Keywords

This view has been locked by amendment(s)

Lay Summary and Keywords

Please provide the following information about your study.

1.0 ***Provide a brief lay summary describing this study. (limit 500 words).**
Among persons with Alzheimer's disease and its related dementias (ADRD), disruptive behaviors are frequent. In these challenging clinical situations, it is common for physicians to prescribe antipsychotic medications, despite limited evidence for the efficacy of these medications for patients with ADRD, and extensive empiric data showing they are dangerous. Building upon an NIA R01-funded project to implement a pragmatic trial of a behavioral economics-based electronic health record (EHR) intervention to reduce inappropriate pre-operative testing for patients undergoing cataract surgery, we will leverage that infrastructure to determine whether a similar EHR intervention can successfully reduce prescribing of antipsychotic medications and total pill days

prescribed for patients with ADRD.

This quality improvement initiative will integrate a “nudge” for the physician ordering any antipsychotic medication for patients with ADRD. The nudge will default the order to the lowest pill dosage, reference academic literature that shows potential harms to ADRD patients who take antipsychotics, and encourage the provider to refer the patient and his/her caregiver to educational materials.

Additionally, there will be an educational component to the intervention. We will send an email to UCLA Health providers who have been randomized to the intervention arm before the start of the intervention. The email will alert them to this intervention (the BPA and the non-pharmacologic educational materials).

To determine the impact of this EHR nudge, we will randomize UCLA physicians who care for ADRD patients, and compare total pill days supplied (primary outcome) and the rate of prescribing antipsychotics for providers who receive the nudge versus those who do not.

2.0 ***List three to five keywords describing this study (separate the words with commas). The keywords may be used for identifying certain types of studies.**

Quality Improvement, Electronic Health Records, Alzheimer's Disease and Related Dementia (ADRD), Low Value Care

3.0 *** Is this study conducted or supported by HHS (e.g., the National Institutes of Health, Centers for Control and Prevention, etc.)?**

(●) **Yes** ( ) No

3.1 *** Is NIH the HHS agency supporting or conducting the study?**

(●) **Yes** ( ) No

3.2 *** Please choose one:**

(●) **I acknowledge that my study is automatically covered by a Certificate of Confidentiality and I understand the responsibilities associated with that Certificate.**

( ) The NIH Certificate of Confidentiality policy does not apply to my study (see guidance at right and explain below)

4.0 *** Is this study regulated by the Food and Drug Administration (FDA)?**

( ) Yes (●) **No**

4.1 **If yes, check all that apply:**

- [ ] Human Drugs
- [ ] Medical Devices
- [ ] Biological Products
- [ ] Mobile Medical Applications
- [ ] Food Additives
- [ ] Color Additives
- [ ] Other

Methods/Procedures - Descriptors

Note: The items listed below are not an inclusive list of methods and procedures that may be used in research studies. The list only includes items that will trigger additional questions related to the research or are needed for the review process

1.0 ***Indicate all that apply to this study.**

- [ ] Audio, Visual or Digital Recordings
- [ ] Certificate of Confidentiality for research not supported by NIH
- [x] **Clinical Trial of a Drug, Biologic, Device or a Behavioral Intervention**
- [ ] Community Based Research
- [ ] Controlled Substances (Schedule I or II)
- [x] **Deception or Partial Disclosure**
- [ ] Devices/Diagnostics (Note: Submit all HUDs in BruinIRB)
- [ ] Drugs/Biologics/Dietary Supplements
- [ ] Genetic Analyses/Genotyping
- [ ] Human Embryonic Stem Cells and/or Induced Pluripotent Stem Cells
- [ ] Human Gene Transfer/ Recombinant DNA
- [ ] Infectious Agents
- [ ] Non-FDA approved medical equipment used with UCLA hospital patients or research participants that operate under the UCLA Hospital License.
- [ ] Radiation (Standard of Care or Investigational Use of radioactive materials, radiation producing machines or ionizing radiation)
- [ ] Substance Abuse Research (with Medication)
- [ ] Treatment in an Emergency Setting (with request to waive consent)
- [ ] **None of the above**

2.0 ***Will the study require services or resources owned/rented/operated or provided by the UCLA Health System (e.g. clinic and/or hospital visit(s), CTRC, professional medical services, clinical treatment, diagnostics, labs, medical supplies, etc.)?**

Please direct any questions about this to The Financial Coverage & Activation Team at coverageanalysis@mednet.ucla.edu.

○ Yes ◉ **No**

ID: IRB#19-002122 View: NEW 6.1 - Funding and Other Study Characteristics

Funding and Other Study Characteristics

1.0 ***Indicate the funding status for this study**.

◉ **Funded**

○ Application for funding is pending

○ Departmental funding / Self funding / No funding

2.0 ***Check all that apply**:

- [ ] The research will be conducted through the UCLA Clinical and Translational Research Center (CTRC)
- [ ] The study will be supported by or conducted in collaboration with the U.S. Department of Defense (DOD)
- [ ] The study will be supported by or conducted in collaboration with the U.S. Department of Energy (DOE)
- [ ] The study will be supported by or conducted in collaboration with the U.S. Department of Justice (DOJ)
- [ ] The study will be supported by or conducted in collaboration with the U.S. Department of Education (ED)
- [ ] The study will be supported by or conducted in collaboration with the U.S. Department of Protection Agency (EPA)
- [x] **None of the above**

2.1 **If you selected DOD, DOE, DOJ, ED, and/or EPA support/collaboration, please provide your assurances that you will review the additional requirements for research supported by the relevant federal agency.**

Agree [ ]

Note: Please refer to the Federally-Supported Research section of the OHRPP guidance document: Funding Considerations for Federally-Funded and Industry-Sponsored Human Research.

ID: IRB#19-002122 View: NEW 6.2 - Funding - Description

Funding - Description

Based on the response to section 6.1/item1, this study is or will be funded. Please provide the following information.

The Office of Contract and Grant Administration (OCGA) provides the list of funding sources used by webIRB in this section. Please check your OCGA paperwork to find the correct name of the funding source(s) for this study. Identifying the right funding source is important because:

▪ webIRB will auto-populate the designated funding source name on the approval letter for the study. Many funding sources require an accurate identification of their name on the IRB approval letter before they will release funding;
▪ The Office of Research Administration uses data from webIRB to generate funding reports.

Click here for tips on how to find the funding source name in webIRB.

1.0 **Identify the funding source(s).**

If a specific funding source has ended, do not delete it, instead please click Update next to the funding entry and **revise item 1.9.**

**Funding Source**	**Funding Source Information**

	Funding Source	Funding Source Information
View	NIH-NIA NATIONAL INSTITUTE ON AGING	see below

**Name of the Funding Source**	NIH-NIA NATIONAL INSTITUTE ON AGING
**If other, specify**	*No Value Entered*
**UCLA PI named on the grant, contract, subcontract or gift:**	CATHERINE SARKISIAN
**Indicate the type of award:**	Grant
**If other award, specify**	*No Value Entered*
**Indicate the Grant Title:**	Pragmatic Trial of an Electronic Health Record/Behvaioral Economics Intervention to Reduce Pre-operative Testing for Cataract Surgery
**Indicate the Award Number assigned by the funding source:**	R01AG5981-01
**Indicate the description that applies to the source of funding named in the above item. If this is a subcontract, indicate the original source of funding:**	Federal
**If Other, specify**	*No Value Entered*
**Attach a copy of the funding proposal, subcontract, or scope of work.**	**Document Name:** 1R01AG059815-01_sum_statements.pdf<br>**Document Version #:** 0.01
**Does the content of this IRB application differ from the activities described in the attached funding proposal, subcontract, or scope of work?**	Yes
**If yes, describe:**	This IRB application differs from the activities described in the attached funding proposal because this is for the Supplement Award. The study described in this IRB will build upon the infrastructure developed for the parent grant study, but instead of focusing on reducing pre-op testing for patients undergoing cataract surgery, this study will focus on reducing prescribing of antipsychotics for patients with ADRD.
**Check this box to indicate that this specific funding has ended**	No

ID: IRB#19-002122 View: NEW 8.1 - Study Design

Study Design

1.0 *__Check all that apply to the study design.__

☐ **Direct subject contact ONLY** – The research activities involve direct contact with study participants (e.g., collection of data or specimens in person or via internet, phone, mail, etc.)

- [x] **No direct subject contact – None of the research activities involve direct contact with study participants and include only analyses of data, records and/or human biological specimens (e.g., medical record or other record review, study of specimens left over from clinical procedures).**
- [ ] BOTH Direct subject contact AND No direct subject contact – Some of the research activities involve direct contact with study participants and some of the research activities involve analyses of data, records and/or human specimens obtained without contact with participants.

ID: IRB#19-002122 View: NEW 8.3 - Clinical Trial of a Behavioral Intervention, Drug, Biologic or Device

This view has been locked by amendment(s)

Clinical Trial of a Behavioral Intervention, Drug, Biologic or Device

You indicated that this study includes a clinical trial (section 2.3/item 1.0). Please provide the following information

1.0 ***Indicate the type of clinical trial.**

Check all that apply:

- [x] **Randomized**
- [ ] Non-randomized
- [ ] Single Blinded
- [ ] Double Blinded
- [ ] Placebo
- [ ] Sham Control
- [ ] Active/Treatment Control
- [ ] Open Label
- [ ] Crossover
- [ ] Washout Period
- [ ] Dose Escalation
- [ ] Other

1.1 **If you indicated "other", specify.**

2.0 ***Indicate the type of clinical trial:**

- ( ) Pilot/Feasibility
- ( ) Phase I
- ( ) Phase I/II
- ( ) Phase II
- ( ) Phase II/III
- ( ) Phase III
- ( ) Phase III/IV
- ( ) Phase IV
- ( ) Open Label Extension/Rollover
- ( ) Expanded Access (Submit all in BruinIRB)
- (•) **Behavioral**

3.0 ***Indicate the status of registration of registering this trial with ClinicalTrials.gov**

- ○ Registered
- ○ Registration Pending
- ◉ **Not Registered**

4.0 **If the trial is registered, provide the Trial Registration Number:**

ID: IRB#19-002122 View: NEW 8.4 - Deception or Partial Disclosure

This view has been locked by amendment(s)

Deception or Partial Disclosure

You indicated that this study includes deception or partial disclosure (section 2.3/item 1.0). Please provide the following information.

1.0 ***Describe the information that will be withheld from, or misinformation that will be provided to participants.**
This is a pragmatic randomized study. Physicians won't be made aware they are in the study.

2.0 ***Explain why it is necessary to involve deception in the research.**
If physicians were made aware they were in the study, it could influence their response when they are exposed to the intervention (i.e., nudge pop-up alert).

3.0 ***Will participants be de-briefed after study participation?**
◉ **Yes** ○ No

3.1 **If yes, describe the plans for de-briefing study participants after participation.**
Results will be shared across the health system, specifically trough department meetings in various specialties like geriatrics and primary care.

3.2 **Attach a copy of the script or material to be used for the debriefing.**

Document Name	Document Version #
debrief script	0.01

4.0 ***Explain why the research could not practicably be carried out without the alteration of consent:**

- ☑ **the deception or incomplete disclosure is necessary to avoid study bias**
- ☐ the deception or incomplete disclosure is necessary to test a hypothesis that requires the participant's misdirection

ID: IRB#19-002122 View: NEW 9.1 - Not Human Subject Research Screening Items

This view has been locked by amendment(s)

Not Human Subject Research Screening Items

You indicated that data and/or specimens would be obtained for this study without direct contact with human subjects (Section 8.1/Item 2). Please provide the following information.

Federal regulations and UC/UCLA policies require IRB review of research involving human subjects. Please complete this section to determine whether the proposed activities constitute Human Subjects

Research. For activities to qualify as human subject research, the activities must meet the regulatory definitions of "research" and "human subjects."

Important Note: If more than one set of data/specimens will be used for this study, consider these questions for all sets of data/specimens. Example: secondary analysis of an existing coded data set and another data set will be collected specifically for the proposed research. The answer to Question 2 would be "yes".

1.0 ***Funding: Will the activities be supported by Federal funding (e.g., NIH, NSF, DoE, and DoD) that is awarded directly to UCLA?**

(●) **Yes** ( ) No

2.0 ***Were or will the data/specimens be collected specifically for the currently proposed research project?**

( ) Yes (●) **No**

3.0 ***Will you obtain data or specimens from an organization outside UCLA?**

( ) Yes (●) **No**

A Material Transfer Agreement (MTA) is a written contract entered into by a provider and a recipient of research material that governs the transfer of tangible research materials between two organizations. At UCLA, the Technology Development Group (TDG) reviews and approves incoming and outgoing MTAs.

Click here http://tdg.ucla.edu/transferring-research-materials for additional information about how to obtain an MTA.

4.0 ***Is this research covered by FDA regulations?**

( ) Yes (●) **No**

5.0 ***Explain in detail how or why the data/specimens are available to UCLA investigators for research and identify the source and the provider of the data/specimens:**

We will be evaluating existing clinical UCLA electronic health record (EHR) data (medical chart review). Specifically, we will be looking at the impact of our QI project on low-value care (i.e., inappropriate prescribing of anti-psychotics for persons with ADRD). We will also investigate medication substitution to other drug classes.

6.0 ***Will you obtain specimens from UCLA Pathology?**

( ) Yes

( ) No

(●) **Not Applicable - not obtaining specimen**

6.1 **If you indicated in Section 9.1/Item 6.0 that the UCLA PI will obtain specimens from UCLA Pathology, provide your assurance that you will first contact UCLA Pathology to determine whether the specimens are/will be available for your study.**

Agree [ ]

Click here http://pathology.ucla.edu/ for additional information.

7.0 ***Is the provider of the data/specimens the UCLA Investigator or a member of the UCLA study team (e.g., co-investigator) for the proposed research?**

( ) Yes (●) **No**

8.0 ***Do the data and/or specimens include personally identifiable information (PII) or protected health information (PHI)?**

(●) **Yes** ( ) No

8.1 **If no, indicate which of the following apply to the data/specimens.**

Check all that apply:

- [ ] **De-identified** - Neither the UCLA investigator nor the provider(s) of the data/specimens possess identifiers. There is no personally identifiable information (PII) linked to the data/specimens and there is no code or code key that could be used to link the data/specimens, directly or indirectly, to PII.
- [ ] **Coded** - The data/specimens are coded and a key to decipher the code exists and could be used to link PII to the data/specimens.

8.1.1 **If you indicated that the data/specimens are *coded.***

Check all that apply:

- [ ] UCLA investigators **will** have access to the code key.
- [ ] UCLA investigators will not have access to the code key - the data/specimens are coded and the identifiers are maintained by the provider of the data/specimens only. The UCLA investigator or a member of the UCLA study team (e.g., co-investigator) will never receive identifiers from the provider.
- [ ] The key to decipher the code is destroyed by the provider before the research begins.
- [ ] The UCLA investigator and the provider of the data/specimens enter into an agreement prohibiting the release of the key under any circumstances.
- [ ] The data/specimens are from a repository or data management center that prohibits the release of the key to investigators.

9.0 ***Does this study use any other types of data/specimens, in addition to those described above, or have you been advised by the OHRPP that this study qualifies as human subject research?**

(•) **Yes** ( ) No

ID: IRB#19-002122 View: NEW 9.2 - Information about Study Data

Information about Study Data

This information is needed to determine how you will best protect the confidentiality of data.

1.0 ***Indicate all that apply to the study data.**

Check all that apply:

- [x] **Obtained from a medical or clinical record**
- [ ] Created or collected as part of health or mental health care
- [ ] Used to make healthcare or mental healthcare decisions and/or provided to other healthcare professionals
- [ ] Research data will be entered into the participants' medical or clinical record
- [ ] **None of the above**

2.0 ***Is it reasonably foreseeable that the study will collect information that State or Federal law requires to be reported to other officials (e.g., child or elder abuse), ethically requires action (e.g., suicidal ideation), or is a reportable disease?**

( ) Yes (•) **No**

2.1 **If yes, explain below and include a discussion of the reporting requirements in the consent document:**

3.0 ***Indicate if any of the following are being obtained and used without any direct contact with study participants.**

- [x] **Records (Not medical)**
- [ ] Human biological specimens
- [ ] **None of the Above**

4.0 *Indicate all identifiers that may be **accessed or included in the research records** for the study:

- [x] **Names**
- [x] **Dates**
- [x] **Age (if over 89 years)**
- [ ] Postal Address
- [ ] Phone Numbers
- [ ] Fax Numbers
- [ ] E-Mail Address
- [ ] Social Security Number
- [x] **Medical Record Number**
- [ ] Health Plan Numbers
- [ ] Account Numbers
- [ ] License/Certificate Numbers
- [ ] Vehicle ID Numbers
- [ ] Device Identifiers/Serial Numbers
- [ ] Web URLS
- [ ] IP Address Numbers
- [ ] Biometric Identifiers (including finger and voice prints)
- [ ] Facial Photos/Images
- [ ] Any Other Unique Identifier (this does not include the code assigned by the investigator to identify the data)
- [ ] **None of the above**

4.1 **If social security numbers will be collected explain why they are necessary, how they will be used, how they will be protected and how long they will be retained.**

5.0 ***Select all that apply:**

- [x] **The data and/or specimens will be directly labeled with personal identifying information when acquired by the investigator for this research**
- [ ] The data and/or specimens will be labeled with a code that the research team can link to personal identifying information when acquired by the investigator for this research
- [ ] The data and/or specimens will not be labeled with any personal identifying information, nor with a code that the research team can link to personal identifying information when acquired by the investigator for this research
- [ ] The data are restricted use data (A term used in Social-Behavioral research. See guidance on the right.)

5.1 **Indicate how the data will be used when this study is completed.**

Check all that apply:

- [x] **Use for this study**
- [x] **Use for possible future research**
- [ ] Use to create a bank or repository at UCLA
- [ ] Add to existing repository
- [ ] Other

5.1.1 **If Other, specify:**

ID: IRB#19-002122

View: NEW 9.3 - Data Security

This view has been locked by amendment(s)

Data Security

You indicated that the study team will have access to personally identifiable or coded information (Section 9.2/item 5). Please complete the following items.

1.0 ***Do you agree to follow the OHRPP Data Security in Research guidance and procedures?**

- (●) **Yes**
- ( ) I have an alternate equally effective plan (*Note: The plan must be attached to item #2.1*)

2.0 ***Do you have a data security plan for this study?** *(Note: a plan is not required for all studies; it may be recommended in some instance).*

(●) **Yes** ( ) No

2.1 **If yes, attach it here:**

Document Name	Document Version #
ADRD_DSMP	0.01
R01-Data Safety Monitoring Board_ADRD 2019 08 30.docx	0.01

3.0 ***Indicate all that apply to personally identifiable information or codes <u>during conduct of the study</u>:**

- [x] **The data and/or specimens will be coded**
- [ ] The personal identifying information will be removed and destroyed
- [ ] Personally identifying information will be maintained with the data and/or specimens

3.1 **If you indicated that the personal identifying information will be removed or destroyed or that the data/specimens will be coded, provide the following information:**

- **The process for removing and destroying the personal identifying information or for coding the information, and**
- **Indicate who will perform the task**

Each study participant will be assigned a project identification (ID) number, and this ID number will be used rather than names and medical record numbers on all EHR data collected. All analysis of de-identified patient, physician or clinical-level data will take place at UCLA, using password-encrypted servers that can only be accessed first by having unique UCLA network ID's (so must be inside UCLA's firewall). No data will ever be transferred to a hard drive or laptop computer. No attempt will be made by any of the authorized parties using these data to identify individual respondents.

4.0 *__Will coded or personally identifiable data be collected, transmitted or stored via the internet?__

- (●) **Yes**
- ( ) No

4.1 **If yes, indicate all that apply:**

- [ ] A mechanism such as Survey Monkey, Zoomerang, or an e-mail anonymizing service will be used to strip off the IP addresses for data submitted via e-mail.
- [ ] The data will be encrypted.
- [x] **A firewall will be used to protect the research computer from unauthorized access.**
- [ ] Controlled access privileges will be used on the hardware storing the data.
- [ ] Other.

4.1.1 **If you indicated "Other", describe:**

5.0 *__Provide your assurances that if there is a data security breach for this study, the PI will notify the IRB and your department's IT Compliance Coordinator.__

Agree [x]

ID: IRB#19-002122 View: NEW 9.4 - Data Security Plan - During the Study

Data Security Plan - During the Study

You indicated that data and/or specimens for this study will be coded (Section 9.3/item 3). Please complete the following information.

1.0 During the study indicate **how data will be stored and secured** including paper records, electronic files, audio/video tapes, specimens. Specify how the **code key** will be securely maintained, as applicable.

Check all that apply:

1.1 ***Electronic Data**

- [x] **Encryption or password protection software will be used**
- [x] **Secure network server will be used to store data**
- [ ] Stand alone desktop computer will be used to store data (not connected to server/internet)
- [ ] A contracted outside vendor will store the code key. The vendor will have a business associate agreement with UCLA.

- [ ] Other
- [ ] **Not Applicable**

1.2 ***Hardcopy Data, Recordings and Specimens**

- [x] **Locked file cabinet or locked room with limited access by authorized personnel**
- [ ] Locked lab/refrigerator/freezer with limited access by authorized personnel
- [ ] The code key will be kept in a locked file in a locked room
- [ ] The coded data and/or specimens will be maintained in a different room
- [ ] Other
- [ ] **Not Applicable**

1.3 **If you indicated "Other" in item 1.1 or 1.2 above, describe here.**

2.0 *__By checking this box, I provide my assurance that all the person(s) who will have access to the code key have been identified in section 1.1 or section 1.1a.__

Agree [x]

Data Security Plan

You indicated that the study will have access to personally identifiable or coded information (Section 9.2/item 5). Please complete the following items:

1.0 ***After the study is completed**, indicate how the data codes and/or personal identifying information will be handled.

Check all that apply:

- [x] **All data files will be stripped of personal identifiers and/or the key to the code destroyed.**
- [ ] All specimens will be stripped of personal identifiers and/or the key to the code destroyed.
- [ ] Personal identifiers and/or codes linking the data and/or specimens to personal identifiers will be maintained for future research.
- [ ] Audio or Video recordings will be transcribed and then destroyed or modified to eliminate the possibility that study participants could be identified.
- [ ] Photos or Images will be modified to eliminate the possibility that study participants could be identified.
- [ ] Restricted use data will be destroyed or returned to the source.

1.1 **If you indicated that personal identifiers will be maintained for future research, provide the following information:**
a) How the information will be securely handled and stored
b) assure confidentiality, and
c) who will have access to the identifiers and/or codes.

2.0 **Describe any additional steps, if any, to be taken to assure that the subjects' identities and any personal identifying information are kept confidential.**

Use of Data and/or Specimens without Direct Contact

You indicated that some or all of the research activities do not involve direct contact with study participants (Section 8.1/item 1.0). Please provide the following information.

1.0 If **all** of your research activities are without direct contact with study participants, provide the following information:

1.1 **Indicate the purpose of the research, specifying the problems and/or hypotheses to be addressed**:
Because prescribing antipsychotics to persons with ADRD has been demonstrated to have limited efficacy and can be dangerous, the American Geriatrics Society selected avoidance of these dangerous medications as a priority for the Choosing Wisely campaign, and several professional organizations have published guidelines recommending clinicians minimize prescribing these medications in patients with ADRD. There is broad consensus within and outside the medical community that reducing prescribing of antipsychotic medications for older adults with ADRD is an important priority in order to improve quality of life and decrease mortality. Because harms from antipsychotic medications are so common, even a small reduction in prescribing would have a large and meaningful impact on improving important health outcomes (including mortality) for patients with ADRD.

We hypothesize that an interdisciplinary electronic health record (HER)-based quality improvement initiative that applies behavioral economics approaches (i.e., "nudges") will reduce prescribing of antipsychotics in a real-world clinical setting without negatively affecting patients. Reducing exposure of antipsychotic for persons with ADRD is critical to improving patient outcomes, as ADRD patients who take antipsychotic medications experience increased rates of many undesirable side effects including over-sedation, cognitive worsening, falls, strokes, and even death.

This project is fully aligned with UCLA Health leadership's current priority on supporting cross-departmental system change to improve quality of care, outcomes and value for UCLA patients.

1.2 **Describe the study design and proposed data analyses**:
We will integrate a behavioral economics (i.e., "nudge) intervention into the UCLA Health EHR.

We will randomize UCLA Health primary care physicians who care for patients with ADRD to either receiving the EHR nudge (intervention) or not (control). This will be done over a 1-year period and outcomes will be measured at 12 months after the start of the intervention where the main outcomes will be change in total antipsychotic pill days prescribed (primary outcomes) and the change in rate in prescribing of antipsychotics for patients with ADRD.

Secondary patient and physician-level outcomes will include will include the following:
1. Patients: change in rates of falls, emergency department visits, hospitalizations, death.
2. Physicians: perceived change in workflow, autonomy, satisfaction

To ensure we are including only patients that have ADRD, we will conduct a review of 30 patient charts.

1.3 ***If you will conduct genetic analysis with specimens, provide your assurance that the results will not be disclosed to subjects or used for clinical care.**

○ Agree

◉ **Not Applicable**

2.0 ***Describe specimens and/or data that will be acquired without direct contact with study participants. Complete this item for each type used in the study:**

	Source	Data and/or Specimens Information
View	UCLA Electronic Health Records	

**Data and/or Specimens? Indicate all that apply:**	Data
**Indicate whether the data and/or specimens are pre-existing, at the time of this study, and/or if collection will be prospective:**	Pre-existing
**Describe the data and/or specimens and indicate the original collection dates:**	Electronic Health records UCLA Health Records from 2018 to 2022 (end of grant).
**Indicate the approximate number of data records and/or specimens to be collected:**	The number of records collected will depend on the number or procedures or services recorded.
**Will the specimens be used with animals?**	No
**If yes, indicate the IACUC Number:**	*No Value Entered*

3.0 ***If any sources of data and/or specimens are not at UCLA, provide your agreement that the appropriate institutional approvals for release will be obtained** (e.g., IRB approval).

○ Agree

◉ **Not Applicable**

If you plan to send UCLA Health data to third parties, please contact the CTSI for directions about additional requirements. https://www.ctsi.ucla.edu/researcher-resources/pages/third_party

4.0 **Attach any data abstraction tools or lists with the data elements to be collected.**

Document Name	Document Version #
Study Metrics 08.23.2019 (FINAL).pdf	0.01

If you will access information from UCLA Health records, attach a copy of your completed UCLA EHR Data Abstraction template for research. For more information see: https://ctsi.ucla.edu/researcher-resources/pages/datarequests

ID: IRB#19-002122 View: NEW 9.8 - Data and/or Specimens for Possible Future Use

Data and/or Specimens for Possible Future Use

You indicated that prospectively collected data and/or specimens would be stored for future use (Section 9.2/item 5.1). Please provide the following information.

1.0 ***Specify what information directly or indirectly linked to the subject will be provided with data and/or specimens to other investigators.**

Check all that apply:

- [ ] No subject identifiers (The data/specimens are anonymous; no one including the investigator could identify the person from whom the materials were gathered.)
- [ ] The data will be coded (A code links the data/specimens to the study participants. A key to the code exists.)
- [ ] Personal Identifying Information
- [x] **Not applicable, the data will not be shared outside the study team.**

2.0 **Distribution Rules: Describe the criteria used to determine the adequacy of requests to obtain data and/or specimens (e.g., the type of researchers that will be eligible to receive data):**
N/A- Only the current research team will use the de-identified data for research.

ID: IRB#19-002122 View: NEW 11.2 - Characteristics of Study Population

Characteristics of Study Population

1.0 ***Indicate the age range of the study participants.**

Check all that apply:

- [ ] 0 to 6 years
- [ ] 7 to 11 years
- [ ] 12 to 17 years
- [ ] 17 or younger **in California** who can consent for themselves - see note below
- [ ] 17 or younger **outside California** who can consent for themselves - see note below
- [x] **18 years or older**

NOTE:

- For additional information on minors **in California** who are permitted to consent for themselves please refer to the section "Legal Exceptions Permitting Certain Minors to Consent" in the OHRPP Guidance document, Child Assent and Permission by Parents or Guardians
- For additional information on minors **outside of California** who are permitted to consent for themselves please refer to the section "Exceptions Outside of California" in the OHRPP Guidance document, Child Assent and Permission by Parents or Guardians

2.0 ***Indicate if any of the following populations/specimens will be specifically recruited/obtained for the study.**

- [ ] Adults who are competent to give informed consent
- [ ] Adults unable to give informed consent
- [ ] Adults with diminished capacity to consent
- [ ] Fetal Tissue
- [ ] Neonates
- [ ] Participants Unable to Read, Speak, or understand English
- [ ] Pregnant Women/Fetuses
- [ ] Prisoners
- [ ] UCLA Faculty/Staff

- [ ] UCLA Students
- [ ] Wards
- [x] **Unknown/Not Applicable**

3.0 * **Is it possible that there may be non-English speakers enrolled in this study or children whose parents are non-English speaking?**

( ) Yes (•) **No**

ID: IRB#19-002122 View: NEW 14.1 - Risks & Benefits

This view has been locked by amendment(s)

Risks & Benefits

Benefits

1.0 ***Are there any potential direct benefits (physical, psychological, social or other) to study participants?**

( ) Yes (•) **No**

1.1 **If yes, describe.**

2.0 ***Describe the potential benefits to society including the importance of the knowledge to be gained.**

Reducing patient exposure to unnecessary care is central to improving patient outcomes and value. This project is fully aligned with UCLA health leadership's current priority on supporting cross-departmental system change to improve quality of care, outcomes, and value for UCLA patients.

ADRD patients who take antipsychotic medications experience increased rates of many undesirable side effects including over-sedation, cognitive worsening, falls, strokes and even death. While there are a small fraction of clinical situations where the benefits of these medications to patients with ADRD justify the increased risk of harm, there is strong consensus that non-pharmacological approaches are underutilized while overall prescribing rates are far too high. Unfortunately, despite increasing awareness of the harms of antipsychotic medications for patients with ADRD, as many as one-third of U.S. patients with ADRD are still taking prescribed antipsychotic medications. Reducing antipsychotic medication prescribing among older adults with ADRD would substantially improve quality of life for millions of older adults.

Risks

3.0 ***Indicate the potential risks/discomforts, if any, associated with each intervention or research procedure.**

Additionally discuss any measures that will be taken to minimize risks. If data are available, estimate (a) the probability that a given harm may occur, (b) its severity, and (c) its potential reversibility. The information provided should be reflected in risks section of the informed consent documents.

If this is an exempt study and there are no risks, indicate N/A. Otherwise, please see the help text.

In the proposed project, data will be analyzed from UCLA's electronic health records (EHR). The risk to patients is low. The principal risk in the analyses is inadvertent disclosure of protected health information (PHI), which could lead to breaches of patient privacy, identify theft, loss of public trust, among other serious consequences. To avoid this risk, we will use comprehensive data safeguarding procedures as described in the next section.

Risk/Benefit Analysis

4.0 ***RISKS/BENEFIT ANALYSIS: Indicate how the *risks to the participants are reasonable in relation to anticipated benefits*, if any, to participants and the importance of the knowledge that may reasonably be expected to result from the study:**
The project will evaluate a quality improvement initiative. The analysis will not directly benefit patients. However, the actual quality improvement aims to reduce low-value care to patients at UCLA.

Alternatives

5.0 ***Indicate the alternatives to participating in this study.**

Check all that apply.

- [ ] All types of studies - Choose not to participate in the study
- [x] **Clinical/Intervention Studies - Receive standard of care instead of participating in the study**
- [ ] Clinical/Intervention Studies - Medication, device, or other treatment is available off study
- [ ] Item is Not Applicable (e.g., study of existing data)
- [ ] Other

5.1 **If "other" was selected, specify.**

5.2 **If this is a clinical/intervention study:**

Describe the standard of care or activities at UCLA (or study site) that are available to prospective participants who do not enroll in this study. If not applicable to your study, state not applicable (N/A).
Patient will receive standard of care if not participating in the study.

ID: IRB#19-002122 View: NEW 17.1 - HIPAA Authorization

HIPAA Authorization

According to your responses to section 9.2/item 1.0, this study uses protected health information. Please provide the following information.

1.0 ***Indicate all that apply to use of or disclosure of PHI in this study:**

- [ ] All UC participants will sign a UC HIPAA Research Authorization for Release of Personal Health Information for Research.
- [ ] **Another Institutions' Healthcare Authorization** for Release of Health Information will be used **or a waiver** for release of health information will be granted **from another Institution**.
- [ ] **A Waiver of HIPAA Research Authorization** is requested for **screening** using UC medical records. I assure that the PHI collected for this study will not be reused or disclosed, except as indicated in this application.
- [x] **A Total Waiver of HIPAA Research Authorization is requested for the entire study. I assure that the PHI collected for this study from UC records will not be reused or disclosed, except as indicated in this application.**
- [ ] **Limited Data Set with a Data Use Agreement** will be obtained from UC medical records. I assure that I will follow the data security plan outlined in this application to protect the identifiers from improper use or disclosure.
- [ ] **None of the above. This study will be conducted outside the United States**

If you will access information from UCLA Health records, additional permissions are required. These permissions are described at: https://ctsi.ucla.edu/researcher-resources/pages/datarequests

1.1 Please identify the persons who will access UC Health records:

- ◯ CTSI Informatics Program Staff (https://ctsi.ucla.edu/researcher-resources/pages/datarequests)
- ◯ Key personnel listed in Section 1.1/5.0

2.0 *Indicate to whom or where you will grant access to personal identifying information (including PHI) as part of the study process:

- ☑ **There is no plan to share identifiers outside the study team**
- ☐ The study sponsor; on site only (if there is more than one study sponsor, specify below).
- ☐ A foreign country or countries
- ☐ Other

2.1 If you checked "other", "a foreign country or countries", or if "there is more than one sponsor", specify.

3.0 *The investigator's agreement is needed to the following:

- The protected health information requested is the minimum necessary to meet the research objectives

- The protected health information that is obtained as part of this study will not be used or disclosed to any other person other than study personnel or to the parties listed in item Section 17.1/item 2, except as required by law.

- Study Sponsors will **not** be provided with personal identifying information (including PHI) to take from the study site at any time, including the end of the study.

- Data and specimens shared with outside entities, such as study sponsors, will be coded or de-identified.

Agree ☑

ID: IRB#19-002122 View: NEW 17.2 - HIPAA - Waiver of Authorization

HIPAA - Waiver of Authorization

According to your responses to Section 17.1/item 1, a waiver of authorization is requested. Please provide the following information.

In addition to the information that will be requested later in this application for a waiver of informed consent, HIPAA requires the following information for a waiver of authorization:

1.0 *Indicate why the research could not be practicably conducted without access to and use of the protected health information.

Check all that apply.

- ☑ **The PHI is needed to identify potential participants with a specific medical condition**

- [x] **It would not be feasible to individually contact the large numbers of potential subjects in the study**
- [x] **It would not be possible to locate many of the individuals whose records would be used for the study**
- [ ] Many of the individuals, whose records would be used for the study, are now deceased
- [ ] Other

1.1 **If you checked "other", specify.**

ID: IRB#19-002122 View: NEW 20.1 - Informed Consent Process

Informed Consent Process

You indicated that adults (and/or minors who are permitted to consent for themselves) are participating in the study (Section 11.2/item 1.0 or Section 12.2/item 1.0).

For additional information on minors who are permitted to consent for themselves please refer to the section "Legal Exceptions Permitting Certain Minors to Consent" in the OHRPP Guidance document, Child Assent and Permission by Parents or Guardians.

1.0 *__Indicate your plans for obtaining informed consent for this study.__

Check all that apply:

- [ ] **Signed consent** will be obtained from the research participant or Legally Authorized Representative.
  - Signed consent means research participants will be asked to **sign and date** a written consent form.
- [ ] **A waiver of signed consent is requested for the entire study.** One of the following procedures will be conducted:
  - **A written information sheet will** be used. Signed consent will not be obtained from research participants.
  - **Oral consent** will be obtained from the research participant or Legally Authorized Representative (LAR)
  - This option should be selected if the study involves consenting participants via the internet.
- [x] **A waiver of consent is being requested.**
  - **Research participants will not be asked to sign a consent form or give oral consent**
- [ ] Consent will be obtained by a collaborating institution.

1.1 **- If you checked more than one plan above, list the study groups and the plan that you will use for each.**
- If you checked "Consent will be obtained by a collaborating institution", explain the consent process and upload a copy of the most recent approved consent document in item 1.2.

1.2 **If applicable, attach the consent document(s) from collaborating institution(s).**

Document Name	Document Version #
There are no items to display	

Request to Waive Informed Consent for the Study

You indicated that you are requesting a waiver of consent (Section 20.1/item 1). The following information is needed.

1.0 *__Does this study pose more than minimal risk?__

○ Yes ◉ **No**

2.0 *__Would the participants' rights and welfare be adversely affected by waiving consent?__

○ Yes ◉ **No**

3.0 *__Explain why the research could not practicably be carried out without the waiver of consent.__

Check all that apply.

- ☐ It would not be possible to contact all of the participants associated with the data or specimens to obtain consent
- ☐ The design of the study does not allow the possibility of obtaining consent
- ☐ The size of the potential study population is so large it would not be feasible to obtain consent
- ☑ **Requiring informed consent may introduce systematic bias into the data**
- ☐ The risk of contacting the participants is greater than the risk of the study procedures
- ☐ Other

3.1 **If you indicated that the study design does not allow the possibility of obtaining consent, or that requiring consent may introduce systematic bias or checked “other”, provide any information that may assist the IRB to understand why obtaining consent would not be feasible.**

If research staff notified study participants (i.e., providers who are randomized to receive the "nudge" or no) that this was a study to reduce prescribing of certain medications (i.e., antipsychotics) providers might change behavior regardless of the EHR-based "nudge" alert and we would therefore not know if observed changes were from the actual Quality Improvement program (which we are trying to measure) or from our staff giving participants this idea when asking for consent.

4.0 *__Would it be appropriate to provide participants with information about the study after their participation?__

Check all that apply.

- ☐ No, the data will not be stored with identifiers with which to contact the participants
- ☐ No, the information that is found will have no impact on treatment or care
- ☐ No, there is not a feasible mechanism by which to notify participants/respondents
- ☐ No, other
- ☑ **Yes**
- ☐ **Not Applicable** - analysis of secondary data

4.1 **If you checked "no other," specify.**

4.2

If you indicated "yes," indicate the information that would be provided and the mechanism.

The physicians will be debriefed regarding their participation in the study. The results of the study will be presented to geriatric and primary care physicians either at grand rounds or department-level meetings.

ID: IRB#19-002122 View: NEW 24.0 - Additional Information and/or Attachments

This view has been locked by amendment(s)

Additional Information and/or Attachments

1.0 **Attach any other documents that have not been specifically requested in previous items, but are needed for IRB Review.**

Document Name	Document Version #
Draft Email_ADRD_2020 12 30.docx	0.01
DSMP	0.01
Nadia CITI training certificate.pdf	0.01
Nadia GCP Certificate.pdf	0.01

2.0 **If there is any additional information that you want to communicate about this study, include it in the area provided. Note: this section should not be used instead of the standard application items.**

ID: IRB#19-002122 View: NEW 100.0 - Instructions for Study Submission

ID: IRB#19-002122 View: Display - Method Description

Audio, Visual or Digital Recordings

Click "OK" below to return to the SmartForm page where you can select the appropriate response.

ID: IRB#19-002122 View: Display - Method Description

Certificate of Confidentiality for research not supported by NIH

The Certificate of Confidentiality button in this section is only if your study is NOT supported or conducted by NIH but you will obtain a Certificate of Confidentiality (for example, for studies collecting information about illegal drug use).

If you previously checked this box for an NIH-supported study before the policy change, you do not need to change your response here.

Certificates of Confidentiality are issued by the National Institutes of Health (NIH) to protect the privacy of research subjects by protecting investigators and institutions from being compelled to release information that could be used to identify subjects with a research project. Certificates of Confidentiality are issued to institutions or universities where the research is conducted. They allow the investigator and others who have access to research records to refuse to disclose identifying information in any civil, criminal, administrative, legislative, or other proceeding, whether at the federal, state, or local level.

Effective October 1, 2017, NIH has updated its policy for issuing Certificates of Confidentiality for NIH-funded and conducted research. For information about the policy change or about obtaining Certificates for research supported by other agencies, please see https://humansubjects.nih.gov/coc/index.

Click "OK" below to return to the SmartForm page where you can select the appropriate response.

ID: IRB#19-002122 View: Display - Method Description

Clinical Trial of a Drug, Biologic, Device or a Behavioral Intervention

A clinical trial is a research study designed to answer specific questions about medical or behavioral treatments. The trial may be interventional or observational. Interventional studies are those in which the research participants are assigned by the investigator to a treatment or other intervention, and the outcomes measured. Observational studies are those in which individuals are observed and the outcomes are measured by the investigators.

Click "OK" below to return to the SmartForm page where you can select the appropriate response.

ID: IRB#19-002122 View: Display - Method Description

Community Based Research

Click "OK" below to return to the SmartForm page where you can select the appropriate response.

ID: IRB#19-002122 View: Display - Method Description

Controlled Substances (Schedule I or II)

Check here only if you are using a Schedule I or II Controlled substance in this study. Research using Schedule I or Schedule II controlled Substances must be submitted to the Research Advisory Panel of California for review and approval prior to initiation. Research using Schedule III, IV, or V Controlled Substances as a study drug do not require review by the Research Advisory Panel. For further information see: http://ag.ca.gov/research/guide.php o Schedule I Controlled Substances are drugs or substances with a high

potential for abuse, that have no currently accepted medical use in treatment in the United States. Examples of Schedule I Controlled Substances are: heroin, lysergic acid diethylamide (LSD), methylenedioxy-methamphetamine (MDMA), marijuana, and psilocybin. o Schedule II Controlled Substances are drugs or substances with a high potential for abuse, that have a currently accepted medical use in treatment in the United States, or a currently accepted medical use with severe restrictions. Examples of Schedule II Controlled Substances are: fentanyl, methadone, methylphenidate, morphine, and oxycodone. For further information see: http://www.deadiversion.usdoj.gov/schedules/index.html

Click "OK" below to return to the SmartForm page where you can select the appropriate response.

ID: IRB#19-002122 View: Display - Method Description

Deception or Partial Disclosure

Deception includes withholding information about the real purpose of the study or purposely giving subjects false information about some aspect of the research to prevent bias. Some professions, such as the American Psychological Association (APA) have ethical codes regarding the use of deception in research. (See sections 8.07 and 8.08 at http://www.apa.org/ethics/code/index.aspx#807) If deception is included in the study, you must also apply for approval of a waiver of the informed consent process (Section 20.1) in addition to selecting the other consent procedures planned for the study (e.g., written or oral consent).

Click "OK" below to return to the SmartForm page where you can select the appropriate response.

ID: IRB#19-002122 View: Display - Method Description

Devices/Diagnostics (Note: Submit all HUDs in BruinIRB)

A medical device is defined, in part, as any health care product that does not achieve its primary intended purposes by chemical action or by being metabolized. Medical devices include, among other things, surgical lasers, wheelchairs, sutures, pacemakers, vascular grafts, intraocular lenses, and orthopedic pins. Medical devices also include diagnostic aids such as reagents and test kits for in vitro diagnosis (IVD) of disease and other medical conditions such as pregnancy. For further information see: http://www.fda.gov/oc/ohrt/irbs/irbreview.pdf

Click "OK" below to return to the SmartForm page where you can select the appropriate response.

ID: IRB#19-002122 View: Display - Method Description

Drugs/Biologics/Dietary Supplements

- Drug: The term "drug" means: articles recognized in the official United States Pharmacopoeia, official Homoeopathic Pharmacopoeia of the United States, or official National Formulary, or any supplement to any of them; and articles intended for use in the diagnosis, cure, mitigation, treatment, or prevention of disease in man or other animals; and articles (other than food) intended to affect the structure or any function of the body of man or other animals.
- Biologics vs. Drugs: Most drugs consist of pure chemical substances and their structures are known. Most biologics, however, are complex mixtures that are not easily identified or characterized. Biological products differ from conventional drugs in that they tend to be heat-sensitive and susceptible to microbial contamination. This requires sterile processes to be applied from initial manufacturing steps. For more information see: http://www.fda.gov/consumer/updates/biologics062608.html#drugs
- Dietary Supplements are products that are intended to supplement the diet and have one of the following ingredients:
  A vitamin
  A mineral
  An herb or other botanical
  An amino acid
  A dietary substance for use by man to supplement the diet by increasing the total daily intake
  A concentrate, metabolite, constituents, or an extract of combinations of these ingredients.

For additional information see: http://www.foodsafety.gov/~dms/supplmnt.html

Click "OK" below to return to the SmartForm page where you can select the appropriate response.

ID: IRB#19-002122 View: Display - Method Description

Genetic Analyses/Genotyping

Genetic analyses/genotyping include, but are not limited to, studies of inheritable conditions or traits, gene markers or mutations, and pedigrees.

Click "OK" below to return to the SmartForm page where you can select the appropriate response.

ID: IRB#19-002122 View: Display - Method Description

Human Embryonic Stem Cells and/or Induced Pluripotent Stem Cells

Research with human embryonic stem cells (hESC) and related lines requires IRB review under the following conditions: o Clinical research in which human subjects are given hESCs or related products. o When the UCLA research team will have a research related direct interaction or intervention with the cell donors, including donation of blastocysts or gametes for the purpose of creating hESCs,. o Cells provided to the UCLA research team that have identifiers or codes that can be linked back to the donor. Research involving hESC requires review and approval by the ESCRO Committee. For further information see: http://www.stemcell.ucla.edu/research

Click "OK" below to return to the SmartForm page where you can select the appropriate response.

ID: IRB#19-002122 View: Display - Method Description

Human Gene Transfer/ Recombinant DNA

Studies involving gene transfer and/or recombinant DNA require approval of the UCLA Institutional Biosafety Committee (IBC). Human gene transfer is an investigational method for correcting defective genes responsible for disease development through one of the following techniques: o A normal gene may be inserted into a nonspecific location within the genome to replace a nonfunctional gene. o An abnormal gene could be swapped for a normal gene. o The abnormal gene could be repaired through selective reverse mutation, which returns the gene to its normal function. o The regulation of a particular gene could be altered. Recombinant DNA molecules, according to the NIH Guidelines, are defined as either: (i) molecules that are constructed outside living cells by joining natural or synthetic DNA segments to DNA molecules that can replicate in a living cell, or (ii) molecules that result from the replication of those described in (i) above.

Click "OK" below to return to the SmartForm page where you can select the appropriate response.

ID: IRB#19-002122 View: Display - Method Description

Infectious Agents

Studies involving the use of Risk Group 2 or 3 infectious agents (such as bacteria, fungi, parasites, prions, rickettsia, viruses, etc.) require approval of the UCLA Institutional Biosafety Committee (IBC).

Click "OK" below to return to the SmartForm page where you can select the appropriate response.

ID: IRB#19-002122 View: Display - Method Description

Non-FDA approved medical equipment used with UCLA hospital patients or research participants that operate under the

Clinical Engineering is responsible for completing incoming inspections on investigational devices that are used to diagnose, treat or monitor a patient and that are used in the patient care area on site at UCLA, but *not* in other hospitals such as Cedars Sinai, CHLA, or Drew. If a device is FDA and/or testing - laboratory approved for the purpose it was designed, then evaluation is not required of the device. If you have a copy of an inspection report from Clinical Engineering, please attach here. As appropriate, please contact Clinical Engineering at 310-267-9000 to arrange an inspection.

Click "OK" below to return to the SmartForm page where you can select the appropriate response.

ID: IRB#19-002122 View: Display - Method Description

Radiation (Standard of Care or Investigational Use of radioactive materials, radiation producing machines or ionizing radiation)

Note: This includes CT-guided biopsy, fluoroscopy use, etc.; MRI is not included. The radiological procedures included in this study must be described in the SafetyNet system. Please create a new SafetyNet application after submitting this webIRB application to the IRB for review.

Click "OK" below to return to the SmartForm page where you can select the appropriate response.

ID: IRB#19-002122 View: Display - Method Description

Substance Abuse Research (with Medication)

Research for the treatment of controlled substance addiction or abuse that uses any drug (scheduled or not) as treatment, requires the review and approval of the Research Advisory Panel of California prior to initiation. For further information see: For further information see: http://ag.ca.gov/research/guide.php

Click "OK" below to return to the SmartForm page where you can select the appropriate response.

ID: IRB#19-002122 View: Display - Method Description

Treatment in an Emergency Setting (with request to waive consent)

Federal regulations allow certain research activities to be conducted in emergency settings with waiver of informed consent - in the interest of facilitating potentially life-saving and life-enhancing research with protecting the rights and welfare of participants. For further information see: o OHRP Guidance: http://www.hhs.gov/ohrp/humansubjects/guidance/hsdc97-01.htm o FDA Guidance: http://www.fda.gov/oc/ohrt/irbs/except.html

Click "OK" below to return to the SmartForm page where you can select the appropriate response.

ID: IRB#19-002122 View: Display - Method Description

None of the above

ID: IRB#19-002122 View: Specimens and/or data that will be acquired without direct contact with study participants

Specimens and/or Data that will be Acquired without direct contact with study participants

1.1 ***Data and/or Specimens? Indicate all that apply:**

- [x] **Data**
- [ ] Specimens

1.2 ***Indicate the source of the data and/or specimens. If the source is UCLA or a previous study, also indicate the IRB#:**

UCLA Electronic Health Records

1.3 ***Indicate whether the data and/or specimens are pre-existing, at the time of this study, and/or if collection will be prospective. Check all that apply:**

- [x] **Pre-existing**
- [ ] Prospective

1.4 ***Describe the data and/or specimens and indicate the original collection dates. If collection is in progress, indicate the planned end date or "continuing."** (*e.g., academic records for children 6-12 years for the time period between 1995-2005, or tumor samples collected from adults between January 1, 2009 to December 31, 2009*).

Electronic Health records UCLA Health Records from 2018 to 2022 (end of grant).

1.5 ***Indicate the approximate number of data records and/or specimens to be collected.**

The number of records collected will depend on the number or procedures or services recorded.

1.6 **If you indicated that you will be using specimens, provide the following information.**

1.6.1 **Will the specimens be used with animals?**

( ) Yes (•) **No**

1.6.1.1 **If yes, indicate the IACUC Number:**

Title: EHR Intervention to Reduce Antipsychotic Medications Among Older Adults with ADRD

Author: Catherine Sarkisian, MD, MSPH

Data Safety Monitoring Board (DSMB)

UCLA IRB#: 19-007825; ClinicalTrials.gov Identifier: **NCT0485169**

1.0 Participants Safety

1.1 Potential Risks and Benefits for Participants

Potential Risks
In the proposed project, data will be analyzed from the UCLA's electric health records (EHR). The risk to patients is low. The principal risk in the analyses of is inadvertent disclosure of protected health information (PHI), which could lead to breaches of patient privacy, identify theft, loss of public trust, among other serious consequences.

Potential Benefits
Reducing patient exposure to unnecessary care is central to improving patient outcomes and value. This project is fully aligned with UCLA Health leadership's current priority on supporting cross-departmental system change to improve quality of care, outcomes, and value for UCLA patients.

ADRD patients who take antipsychotic medications experience increased rates of many undesirable side effects including over-sedation, cognitive worsening, falls, strokes and even death. Though there are a small fraction of clinical situations where the benefits of these medications to patients with ADRD justify the increased risk of harm, there is strong consensus that non-pharmacological approaches are underutilized while overall prescribing rates are far too high. Unfortunately, despite increasing awareness of the harms of antipsychotic medications for patients with ADRD, as many as one-third of U.S. patients with ADRD are still taking prescribed antipsychotic medications. Reducing antipsychotic medication prescribing among older adults with ADRD would substantially improve quality of life for millions of older adults.

1.2 Adverse Event and Serious Adverse Event Collection and Reporting

Safety Monitoring Definitions
Adverse Event (AE) - Any untoward or unfavorable medical occurrence in a human subject, including any abnormal sign, symptom, or disease, temporally associated with the subject's participation in the research, whether or not considered related to the subject's participation in the research. We will monitor the following AEs:

- Emergency room visits, not requiring hospitalization
- Breach of confidentiality
- Suicidal ideation not requiring intervention

Serious Adverse Event (SAE) is any adverse events that result in the following, which we will monitor:

- Death
- Persistent or significant disability/incapacity

- Inpatient hospitalization or prolongation of existing hospitalization
- Suicidal attempt or ideation requiring intervention

Study-Related – An AE or SAE is considered study related if the PI determines that the AE or SAE to be definitely, probably, or possibly related where possibly related means there is reasonable possibility that the incident, experience, or outcome may have been caused by the procedures or interventions involved in the research. AEs or SAEs judged as remotely related or not related are not considered study related.

Unanticipated Problem or Unexpected AE/SAE – problems or events in which the research places participants or others at a greater risk of harm (including physical, psychological, economic, or social harm) than was previously known or recognized. The term "unanticipated problem" is used in this context because some situations may not have produced an adverse event but is still considered an unanticipated problem (e.g. unsecured or stolen patient data which may not result in an AE). An event is unexpected if it is not described in the package insert of cessation medication, in the study protocol, or in the informed consent document.

<u>SAE/EA Response and Reporting Procedure</u>

1) Research staff becomes aware of AE/SAE (via scheduled reviews of EHR data)
2) Research staff notifies Dr. Sarkisian immediately if the event is an SAE or if immediate psychiatric or medical intervention is required, and within 7 days if the event is an AE.
3) Dr. Sarkisian will conduct EHR review when necessary to gather additional information about the event.
4) Dr. Sarkisian will report AE/SAE to DSMB to aid in determining:
    a. <u>Severity</u>: Mild, Moderate, Severe
    b. <u>Expectedness</u>: Expected, Unexpected
    c. <u>Study Related</u>: Definitely, Probably, Possibly, Remotely, Not Study-Related
5) Project staff documents AE/SAE and DSMB determination in study database. The report will be provided to a NIA Program Officer.
6) Dr. Sarkisian will prepare a report for the local IRB as per local, state, and federal reporting requirements.
7) If a death occurs, Dr. Sarkisian will report it to the DSMB and NIA Program Officer within 48 hours of knowing about the event.
8) DSMB will aid in planning measures to prevent future occurrences, if any are warranted.
9) Dr. Sarkisian will make changes to protocol and/or consent form if needed.

To enhance monitoring and study oversight, DSMB and study staff will meet at a minimum of every six months.

<u>1.3 Protection Against Study Risk</u>

<u>Informed Consent Process</u>

In the proposed study, informed consent will not be collected due to the possibility of introducing systematic bias into the data.

If research staff notified potential participants that this was a study to reduce unnecessary lab tests, participants might ask their doctors to have fewer lab tests. Then we would not know if observed changes were from the actual Quality Improvement program (which we are trying to measure) or from our staff giving participants this idea when asking for consent (which would be a source of bias).

After participants have completed the study, they will be debriefed regarding their participation in the study. The results of the study will be presented to the physicians who treat ADRD patients at either a grand rounds or department meeting(s).

Protection Against Risks

To avoid the risk of inadvertent disclosure of PHI, we will use three principle data safeguarding strategies. **(1)** All study personnel will be educated about general principles of subject access, informed consent, confidentiality, data safeguarding, and privacy requirements, and will complete an on-line course required by the UCLA Institutional Review Board. **(2)** Each study participant will be assigned a project identification (ID) number, and this ID number will be used rather than names and medical record numbers on all EHR data collected. All analysis of de-identified patient, physician or clinic-level data will take place at UCLA, using password-encrypted servers that can only be accessed first by having unique UCLA network ID's (so must be inside UCLA's firewall). No data will ever be transferred to a hard drive or laptop computer. No attempt will be made by any of the authorized parties using these data to identify individual respondents. **(3)** Once all peer-reviewed publications using EHR are completed and published, the de-identified data will be destroyed using UCLA IRB protocols for appropriate data removal.

2.0 Interim Analysis

Adverse outcomes including overnight hospital admissions, ED visits, and death will be compared every 4 months among patients who receive the intervention and those who do not. If frequencies of adverse events are found to be statistically significantly higher ($p<0.05$) in the intervention group than the control group, Dr. Sarkisian will report findings to the IRB and DSMB, and serious consideration will be given to stopping the study.

3.0 Data and Safety Monitoring

As part of data management, Dr. Sarkisian will be responsible for ensuring participants' safety on a daily basis. The DSMB will act in an advisory capacity to the NIA Director to monitor participants' safety; evaluate the progress of the study; and to review procedures for maintain the confidentiality of data, the quality of data collection, management, and analyses.

3.1 Frequency of Data and Safety Monitoring

Data monitoring will be performed on a weekly basis by Chad Villaflores (Project Analyst) to maintain data integrity. As data is entered into the system, Chad Villaflores will perform weekly checks in all of the clinical data sets for recurrent missing documentation, data inaccuracies, errors in submitted data and missing data. Such data problems will be sent to the study staff for corrections. Logs of these data issues will be maintained to identify problem areas with specific variables, or with specific study teams allowing Dr. Sarkisian and the DSMB to proactively modify the data collection instruments or re-train study coordinator/data entry staff. Logs of communications with study staff with regard to data cleaning and management will also be maintained to keep track of corrected issues.

The DSMB and Dr. Sarkisian will meet twice annually—either in-person or by teleconference call—to review study progress, data quality and participants' safety. Safety reports will be sent to the NIA Project Officer twice a year and will include a detailed analysis of study progress, data and safety issues.

3.2 Content of Data and Safety Monitoring Report

A progress report will be completed on regular basis, which will include study status, demographics, AS/SAE events, and data errors.

3.3 DSMB Membership and Affiliation
The following individual(s) has/have accepted position(s) as part of the study's DSMB. DSMB membership will be reviewed and approved by NIA. Should there be any questions regarding the independence of the DSMB, it will be addressed and corrected if necessary at that time.

Catherine Chen, MD, MPH
Assistant Professor, Department of Anesthesia and Perioperative Care, University of California, San Francisco

Malaz Boustani, MD, MPH
Richard M Fairbanks Professor in Aging Research, Indiana University

John Boscardin, PhD
Director of the Statistical Laboratory for the Division of Geriatrics and for SFVAMC Health Services Research, University of California, San Francisco

3.4 Conflict of Interest for DSMB's
DSMB should have no direct involvement with the study investigators or intervention. Each DSMB member will sign a Conflict of Interest Statement which includes current affiliations, if any, with pharmaceutical and biotechnology companies (e.g., stockholder, consultant), and any other relationship that could be perceived as a conflict of interest related to the study and/or associated with commercial interest pertinent to study objectives.

3.5 Protection of Confidentiality
Data with PHI will only be shared with members of the research team that are authorized to view such data as part of research procedures. De-identified data will be presented at open sessions of DSMB meetings. Participants' identities will not be known to the DSMB members and all data will be treated as confidential.

3.6 DSMB Responsibility

- Review the research protocol, informed consent documents and plans for data safety and monitoring.
- Evaluate the progress of the trial, including periodic assessments of data quality and timeliness, recruitment, accrual and retention, participant risk versus benefit, performance for the trials sites, and other factors that can affect study outcome.
- Consider factors external to the study when relevant information becomes available, such as scientific or therapeutic developments that may have an impact on the safety of the participants or the ethics of the trial.
- Review study performance, make recommendations and assist in the resolution of problems reported by Dr. Sarkisian.
- Protect the safety of the study participants.
- Report to NIA on the safety and progress of the trial.
- Make recommendations to the NIA and the Principal Investigator concerning continuation, termination or other modifications of the trial based on the observed beneficial or adverse effects of the treatment under study.
- If appropriate, review interim analyses in accordance with stopping rules, which are clearly defined in advance of data analysis and have the approval of the DSMB.
- Ensure the confidentiality of the study data and the results of monitoring.
- Assist the NIA by commenting on any problems with study conduct or enrollment.

1.0 PARTICIPANT SAFETY

1.1 POTENTIAL RISKS AND BENEFITS FOR PARTICIPANTS

Potential Risks:

In the proposed project, data will be analyzed from UCLA's electric health records (EHR). The principal risk in to participants in this project is of inadvertent disclosure of protected health information (PHI), which could lead to breaches of patient privacy, identify theft, and loss of public trust. To avoid this risk, we will use comprehensive data safeguarding procedures as described in the next sections.

Potential Benefits:

There is broad consensus within and outside the medical community that reducing prescribing of antipsychotic medications for older adults with ADRD is an important priority in order to improve quality of life and decrease mortality. Because harms from antipsychotic medications are so common, even a small reduction in prescribing would have a large and meaningful impact on improving important health outcomes for patients with ADRD. Furthermore, because our health system uses Epic (the EHR used by over half of health systems in this nation), a successful intervention could be easily disseminated across the country through the recently launched Epic "App Orchard," with the potential to dramatically decrease falls, stroke, and mortality for millions of Americans with ADRD.

1.2 ADVERSE EVENT/SERIOUS ADVERSE EVENT/UNANTICIPATED PROBLEM COLLECTION AND REPORTING

Because ADRD patients who take antipsychotic medications experience increased rates of many adverse events including over-sedation, cognitive worsening, falls, strokes, and even death, we would expect adverse events to decrease with our intervention.

To monitor changes in these serious events, we will extract from the EHR the following adverse events on a monthly basis: (1) falls, (2) strokes, and (3) death.

Every 6 months, we will summarize rates of these events and report these to the UCLA IRB, the SO, and NIA program officer. Any deaths that occur will be reported within 24 hours to the UCLA IRB, the SO and NIA program officer.

1.3 PROTECTION AGAINST STUDY RISKS

Data Safeguarding Procedures: Dr. Sarkisian will maintain overall responsibility for data safeguarding of all data provided for this project. To avoid the risk of inadvertent disclosure of

PHI, we will use three principle data safeguarding strategies. (1) All study personnel will be educated about general principles of subject access, informed consent, confidentiality, data safeguarding, and privacy requirements, and will complete an on-line course required by the UCLA Institutional Review Board. (2) Each study participant will be assigned a project identification (ID) number, and this ID number will be used rather than names and medical record numbers on all EHR data collected. All analysis of de-identified patient, physician or clinic-level data will take place at UCLA, using password-encrypted servers that can only be accessed first by having unique UCLA network ID's (so must be inside UCLA's firewall). No data will ever be transferred to a hard drive or laptop computer. No attempt will be made by any of the authorized parties using these data to identify individual respondents. (3) Once all peer-reviewed publications using EHR are completed and published, the de-identified data will be destroyed using UCLA IRB protocols for appropriate data removal.

Protection of Risk: Each study participant will be assigned a project identification (ID) number, and this ID number will be used rather than names and medical record numbers on all EHR data collected. All analysis of de-identified patient, physician or clinic-level data will take place at UCLA, using password-encrypted servers that can only be accessed first by having unique UCLA network ID's (so must be inside UCLA's firewall). No data will ever be transferred to a hard drive or laptop computer. No attempt will be made by any of the authorized parties using these data to identify individual respondents.

2.0 INTERIM ANALYSIS

We do not plan any interim analyses.

3.0 DATA AND SAFETY MONITORING

The Principal Investigator (PI) Dr. Sarkisian will be responsible for ensuring participants' safety on a daily basis. The *Data and Safety Monitoring Board (DSMB)* will act in an advisory capacity to the NIA Director to monitor participant safety, evaluate the progress of the study, to review procedures for maintaining the confidentiality of data, the quality of data collection, management, and analyses.

3.2 CONTENT OF DATA AND SAFETY MONITORING REPORT

Safety reports are sent to the SO twice a year and will include a detailed analysis of study progress, data and safety issues.

3.3 DSMB MEMBERSHIP AND AFFILIATION

Please see second attachment for detailed information about the DSMB.

3.4 CONFLICT OF INTEREST FOR DSMB'S

Once approved by NIA, the DSMB members will sign a Conflict of Interest Statement that will include current affiliations, if any, with pharmaceutical and biotechnology companies (e.g., stockholder, consultant), and any other relationship that could be perceived as a conflict of interest related to the study and / or associated with commercial interests pertinent to study objectives.

3.5 PROTECTION OF CONFIDENTIALITY

Data will be presented in a blinded manner in SO reports. In SO reports, data and discussion are confidential. Participant identities will not be known to the SO.

3.6 DSMB RESPONSIBILITIES

The DSMB Charter provides a detailed list of the DSMB/ SO responsibilities. They include:

- Review study performance, make recommendations and assist in the resolution of problems reported by the Principal Investigator
- Protect the safety of the study participants
- Ensure the confidentiality of the study data and the results of monitoring
- Assist the NIA by commenting on any problems with study conduct, enrollment, sample size, and/or data collection
